# Synthetic promoter design in plants: integration of computational and experimental approaches

**DOI:** 10.3389/fpls.2026.1768521

**Published:** 2026-02-13

**Authors:** Anna E. Yaschenko, Jose M. Alonso, Anna N. Stepanova

**Affiliations:** Department of Plant and Microbial Biology, Genetics and Genomics Academy, North Carolina State University, Raleigh, NC, United States

**Keywords:** chromatin accessibility, *cis*-element, core promoter, enhancer, gene expression regulation, transcription factor binding site

## Abstract

Understanding how to engineer transcriptional regulation in plants is key to advancing both fundamental knowledge and practical applications in plant biology. Native gene promoters, while widely used, are constrained by evolutionary pressures that limit their modularity, tunability, and predictability across genetic backgrounds and species. Synthetic promoters, artificial DNA sequences composed of defined cis-regulatory elements (CREs) for recruitment of gene-specific transcription factors (TFs) and general transcriptional machinery, provide a powerful alternative for achieving fine-tuned transcriptional control. This review examines the design and application of synthetic promoters in plants, emphasizing current strategies, ongoing challenges, and avenues for innovation. We cover the structure of plant promoter architecture, including the contributions of core, proximal, and distal regions, and highlight how promoter grammar (i.e., motif identity, motif distance from transcription start site, spacing between motifs, helical phase of TF binding, motif orientation, and combinatorial interactions between motifs) impacts transcriptional activity. We outline how synthetic promoters are designed and validated via high-throughput reporter assays. Applications of synthetic promoters are discussed across functional genomics studies, biosensor creation, logic gate-based genetic circuits, and practical crop engineering, with examples covering constitutively expressing, hormone-responsive, pathogen-inducible, and abiotic stress-responsive promoter designs. We discuss traditional and emerging computational frameworks that enable CRE identification, novel synthetic promoter generation, and prediction of promoter sequence activity *in silico* to inform the rational design of promoters with predictable performance and spatiotemporal expression. We emphasize the importance of integrating experimental studies and computational approaches through iterative Design-Build-Test-Learn (DBTL) cycles to standardize and optimize frameworks for synthetic promoter development. By combining insights from plant promoter studies with advances in both plant-specific and non-plant synthetic promoter generation and computational modeling, researchers can expand synthetic promoter libraries to enable complex man-driven transcriptional regulation across various plant systems.

## 1 Introduction

The ability to control gene expression precisely in plants has emerged as a central goal of modern plant biotechnology. Gene expression is regulated by a multilayered network of epigenetic, transcriptional, post-transcriptional, translational, and post-translational processes, with transcriptional regulation viewed as one of the most important components determining spatial, temporal, and quantitative expression. Transcriptional regulation is mediated through interactions between short DNA motifs, referred to as *cis*-regulatory elements (CREs), found predominantly within the promoters of genes, and the transcription factors (TFs) that bind them, orchestrating when, where, and how strongly a gene is expressed (Marand et al., 2023). TFs generally bind CREs in order to initiate the process of transcription, particularly through their activation domains that enable recruitment of transcriptional machinery (e.g. RNA polymerase II and its associated general TFs), though in some cases, TFs may repress transcription instead (Lu and Lionnet, 2021). These CREs, commonly referred to as transcription factor binding sites (TFBS), can be arranged in various configurations within a promoter, from tandem homotypic or heterotypic sites associated with one TF or TF family, to overlapping or tandem sites associated with various TFs. Throughout time, promoters have evolved to contain various types of TFBS and other CREs to enable differential gene expression across tissues, developmental stages, and environmental contexts, resulting in dynamic landscapes of gene expression throughout an organism. These interactions become especially complex in plants due to their sessile nature and, accordingly, the need to rapidly reprogram gene expression in response to fluctuating conditions (e.g. drought, pathogen attack, or nutrient limitations) (Cheong, 2023). As a result, modulating promoter architecture and sequence composition is a mechanism through which plants can achieve phenotypic plasticity through transcriptional regulation. Dissecting the interactions of TFs and CREs across various promoter regions is not only necessary to understand the molecular mechanisms behind plant development and adaptation to ever-changing environmental conditions, but also to develop strategies for engineering robust gene expression systems in plants.

Precise control of transcription is essential in both foundational and applied research contexts, as indiscriminate or leaky expression can obfuscate findings by generating off-target phenotypes or compromising the fitness of the host. Historically, natural promoters have been utilized to modulate transgene expression (Brooks et al., 2023). Although natural constitutive, inducible, and tissue-specific natural promoters have been described, these promoters often come with limitations (Potenza et al., 2004). Constitutive expression may negatively impact plant health, as global overexpression of some genes can be detrimental to survival or fecundity (Potenza et al., 2004). Inducible promoters that are native to plants may behave unexpectedly, as the compounds utilized to induce them are endogenously regulated (Potenza et al., 2004). Natural tissue-specific promoters are often expressed in several parts of the plant and may possess leaky activity, making it difficult to drive target genes in desired expression patterns (Potenza et al., 2004). Moreover, promoter activity can vary across genetic backgrounds or species, a phenomenon driven by differences in the availability and concentration of endogenous TFs and the local chromatin accessibility landscape across various host organism tissues (Strader et al., 2022). As a result, researchers often lack promoters that combine the desired degree of both spatial and temporal, as well as quantitative, control of target gene expression (Yaschenko et al., 2022). These limitations highlight the pressing need for promoter engineering strategies that move beyond leveraging natural sequences and into rationally designed, synthetic promoters tailored for specific applications.

Though not from plants, the *nopaline synthase* (NOS) promoter from *Agrobacterium tumefaciens* and the cauliflower mosaic virus (CaMV) *35S* promoter are both widely used in plant research as constitutive promoters (Ali and Kim, 2019; Goring et al., 1991). The *35S* promoter was originally characterized as a strong constitutive promoter that drives viral gene expression in most plant tissues (Odell et al., 1985). Since then, it has become the most well-known and commonly employed promoter in plant biotechnology. Both the full *35S* promoter sequence and the core sequence of this promoter are frequently utilized, with the former serving as a high-expressing constitutive promoter across many plant species, and the latter functioning as a minimal (core) promoter that has the minimum elements necessary for transcription initiation, making it ideal for synthetic promoter construction (Ali and Kim, 2019). Another commonly employed promoter in plant synthetic biology is the *UBIQUITIN10* (*UBQ10*) promoter, derived from *Arabidopsis thaliana*, which provides constitutive expression in most tissues and developmental stages (Kumar et al., 2022; Norris et al., 1993). Its constitutive nature has made *UBQ10* a feasible alternative to *35S*, especially in conditions where viral promoters are less effective or undesirable.

However, reliance on native and heterologous promoters limits the realm of possible expression. Native promoters are products of evolutionary selection and are thus restricted by designs that were advantageous for survival and fecundity, meaning that there are some patterns of expression that cannot be derived from promoters of native genes. In contrast, synthetic promoters do not have those evolutionary constraints and could, in principle, offer a wider range of modularity and tunability, enabling precise control of expression levels and spatiotemporal domains. These properties make synthetic promoters crucial resources for studies in functional genomics, general plant engineering, and biosensor design in plants (Yaschenko et al., 2022). Artificially constructed DNA sequences that integrate native or novel CREs in defined specific arrangements to drive transcription under particular conditions, such as the auxin-responsive DR5 promoter, have been employed for decades to specify environmental responsiveness and tune domains of expression (Ulmasov et al., 1997; Ali and Kim, 2019). By utilizing these sequences as promoters to drive genes of interest, researchers can generate patterns of target gene expression that are inducible, tissue-specific, and/or developmentally regulated.

Through motif engineering, researchers can build promoters responsive to hormones like ethylene or stresses such as drought and salinity (Fernandez-Moreno et al., 2025; Ali and Kim, 2019). This is achieved by combining CREs in various arrangements to artificially recruit native TFs, granting researchers control of the strength and specificity of target gene expression. This modularity allows synthetic promoters to integrate multiple signals and produce specific outputs, effectively programming plants with synthetic promoter-driven genetic circuits to respond to complex environmental conditions.

Though synthetic promoters are incredibly useful, they do not come without their limitations. Most synthetic promoters still rely on the existence of native TFs and CREs, requiring in-depth knowledge of TF binding preferences and expression landscapes, as well as CRE-conferred expression profiles, if we hope to accurately leverage native TFs to drive specific expression. In this review, we analyze current design and applications of synthetic promoters in plants, highlighting how these promoters can be utilized in gene regulation and functional studies. We first review the components of transcriptional regulation, including the role of promoters, classification of TFs, and associated CREs, before examining current synthetic promoter design guidelines based on what is known about promoter architecture and grammar. We then highlight the employment of synthetic promoters as tools for fine-tuned control of gene expression in both foundational and applied research. We provide a non-comprehensive overview of computational tools and databases available for promoter activity prediction and design. We analyze the integration of computational design and predictive modeling with traditional experimental methods in plant synthetic biology to guide the process of designing optimized synthetic promoters that can achieve tunable, specific expression in plants. Finally, we address current limitations and challenges in synthetic promoter employment, finishing off with potential avenues that can be leveraged for more robust synthetic promoter design. The goal of this review is to provide a broad overview of the topic of synthetic promoter development and highlight the importance of integrating computational approaches with existing experimental validation methods to streamline rational synthetic promoter engineering.

## 2 General transcriptional regulation in plants

**Figure 1:**
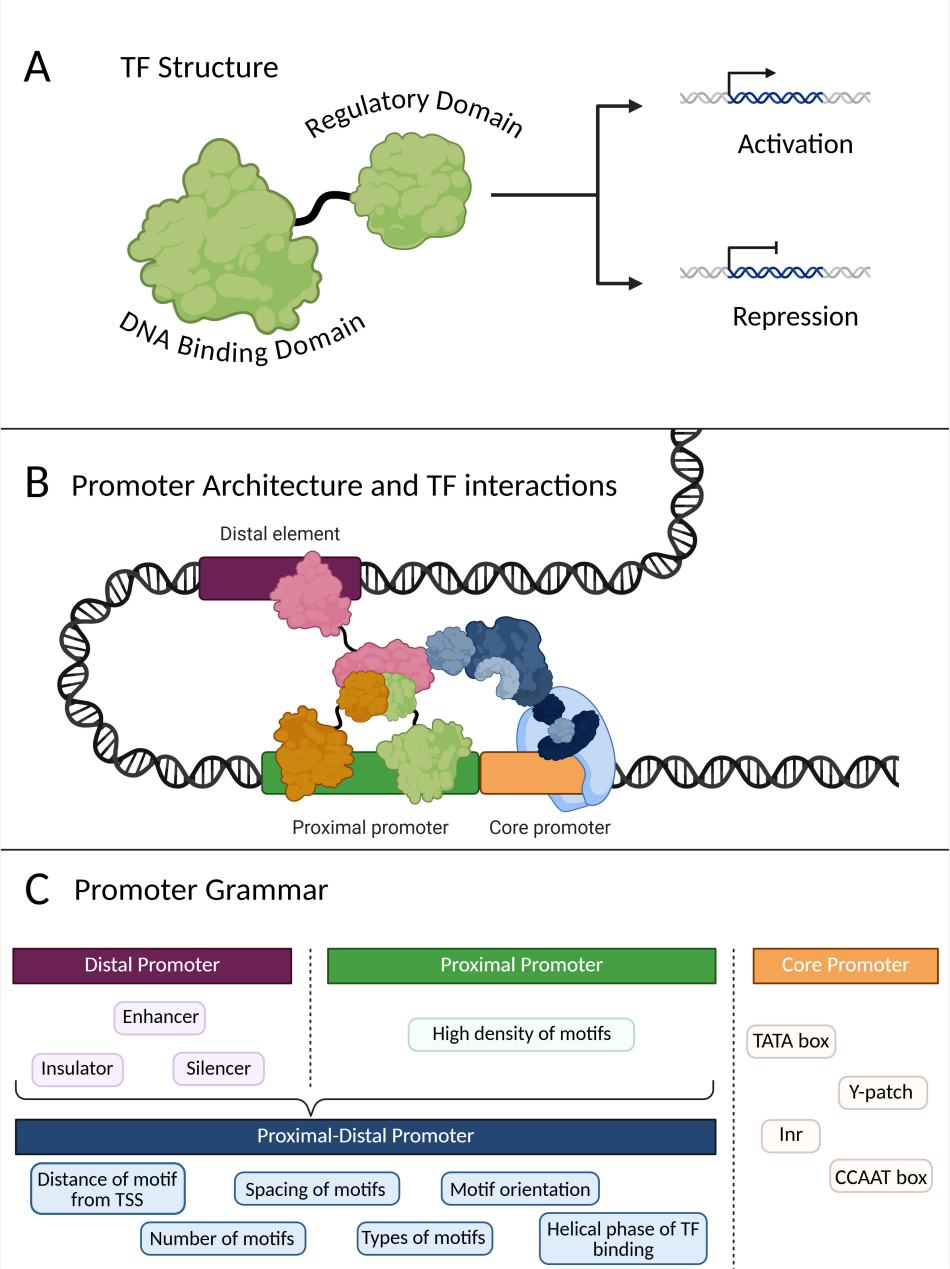
Overview of TFs and promoters. **(A)** Schematic of the different parts of a TF. Regulatory domains can either lead to activation or repression of target gene transcription. **(B)** Schematic depicting potential interactions between TFs binding to distal, proximal, and core promoters. Pink TF binds to a distal element while orange and green TFs bind to the proximal promoter region. Their regulatory domains then interact to facilitate recruitment of general transcriptional machinery (in blue). DNA looping has to occur for the regulatory domains of all TFs to interact, particularly as it relates to the distal promoter. **(C)** Schematic depicting different characteristics of distal, proximal, and core promoters. The distal promoter can have motifs that act as enhancers, silencers, or insulators of transcriptional activation. The proximal promoter generally has the highest density of motifs, and the core promoter can contain motifs encompassing basic transcriptional cis-elements such as the TATA box, Y-patch, Inr, and the CCAAT box. Motifs in proximal promoters may be affected by various promoter “grammar” characteristics, including distance, spacing, orientation, type, and copy number. Additionally, some plant genes may possess downstream promoter elements (DPE) in the 5’ UTR. Created in https://BioRender.com.

Transcriptional regulation in plants involves a coordinated sequence of events rooted in TF-DNA interactions. TFs are modular proteins, typically composed of one or more DNA-binding domains (DBDs) that recognize and bind to specific CREs, and regulatory domains that recruit or interact with transcriptional machinery, co-factors, and chromatin remodelers (**Figure 1A**). The separation of binding and regulatory modules allows TFs to be flexible in their function. A TF may act as an activator in one context and as a repressor under different conditions, depending on interacting partners (**Figure 1A**).

Promoters are central to this type of regulation, as they contain binding sites for gene-specific TFs that can activate or repress transcription and binding sites for the basal transcriptional machinery to initiate transcription of a given gene (**Figure 1B**). Transcription initiation relies on the recruitment and formation of the pre-initiation complex (PIC), which is composed of RNA polymerase II (Pol II) and a host of general transcription factors (GTFs), including TFIIA, TFIIB, TFIIE, TFIIF and TFIIH. Additionally, the PIC assembly includes TFIID, which consists of the TATA-binding protein (TBP) and a variable number of TBP-associated factors (TAFs) depending on species (Freytes et al., 2024). This assembly allows for the proper positioning of Pol II at the transcription start site (TSS). However, transcription initiation *in vivo* cannot occur without the Mediator, a multisubunit complex that bridges gene-specific TFs and the PIC (Freytes et al., 2024). In plants, the Mediator has been found to play a very important role in various stress responses, as the subunits that make up the Mediator complex can influence expression through recruitment of additional transcriptional regulators (Samanta and Thakur, 2015). Once the Mediator relays gene-specific TF signals to the PIC and Pol II is properly positioned, transcriptional elongation can occur, followed by termination of transcription.

Terminators, given that they are required for the process of terminating transcription, as well as proper maturation of the 3’ end of pre-mRNA, also play a prominent role in gene transcription, working together with promoters to determine transcriptional dynamics *in planta* (de Felippes and Waterhouse, 2022). Certain terminators have been found to increase or decrease transcription in combination with specific promoters (Brooks et al., 2023). Though researchers have historically leveraged bacterial and viral terminators for use in plants, recently, the plant research community has invested a great deal of time into characterizing native plant terminators and testing *in silico* evolved synthetic terminators that can be employed in combination with synthetic promoter sequences to modulate transcription (Gorjifard et al., 2024). It is important to consider terminator selection alongside promoter design, as the specific paired promoter-terminator combination can substantially influence overall transcriptional behavior and ultimately shape the effectiveness of synthetic promoter-driven constructs.

Chromatin state and accessibility, nucleosome positioning, histone modifications, and DNA methylation further modulate whether TFs and general transcriptional machinery can access promoters (Chen et al., 2024). Chromatin containing a gene of interest can either be in a euchromatic (open) state, leading to the possibility of active transcription, or a heterochromatic (condensed) state, which leads to gene silencing. Therefore, considering the effects of epigenomic factors is necessary when attempting to understand the nature of transcriptional outcomes in plants.

## 3 Transcription factor classification

Understanding TF classification provides necessary context when engineering synthetic promoters, as TF family identity is linked to DNA-binding specificity and consequently informs rational selection of CREs utilized in promoter construction. A substantial portion of plant genomes codes for TFs. In *Arabidopsis thaliana*, a little over 5% of genes encode TFs, corresponding to over 1,500 proteins (Riechmann and Ratcliffe, 2000). Comparative genomics studies have shown that TF families expand more rapidly in plants than in animals, likely due to plants’ sessile lifestyle and their reliance on transcriptional plasticity for survival (Shiu et al., 2005). Several major TF families in plants are shared with animals, including MYB, bHLH, bZIP, and MADS (Lehti-Shiu et al., 2017). However, many families are unique to plants, including AP2/ERF, NAC, WRKY, and ALOG (Riechmann and Ratcliffe, 2000; Rieu et al., 2024). These plant-specific TF families play critical roles in plant development, hormone signaling, and stress responses. For example, WRKY TFs regulate pathogen defense via binding to the W-box motif (TTGACC/T), while bZIP proteins regulate stress and light responses through ACGT-containing elements such as the G-box (Birkenbihl et al., 2017; Lindbäck et al., 2023). Many TF families show conservation of binding motifs across plant lineages (Zenker et al., 2025). For example, the G-box motif is recognized by bZIP proteins in both monocots and dicots, while MADS TFs share conserved CArG-box binding preferences across flowering plants (Lindbäck et al., 2023; Zhang et al., 2024). The conservation of TF-TFBS pairings suggests that these relationships can be leveraged in designing synthetic promoters that function similarly across species.

Generally, TFs are classified into families based on the domains they are composed of, particularly their DBDs. However, TF classification into families versus superfamilies or subclasses can often be somewhat obscure, as similar domains may evolve divergent binding specificities. To simplify this process, researchers have developed a structural classification framework for plant TFs by studying the 3D structures of 56 identified plant TF types, with 50 of those TF types fitting into nine superclasses (Blanc-Mathieu et al., 2024). This framework, referred to as Plant-TFClass, provides a simplified classification method that follows mammalian TFs classification practices, with an organizational structure that classifies TFs hierarchically into superclasses, classes, and families (Wingender et al., 2013). The drawback to this method is that it requires 3D structures of plant DBDs, a data source that is only now beginning to expand thanks to recent advancements in accurate protein structure prediction and resolution of protein-DNA interactions. However, it is forward-thinking to employ Plant-TFClass in classifying plant TFs, as some existing databases utilize this framework to classify TF binding profiles, such as the manually curated and regularly updated JASPAR database that has expanded into plant TFs (Rauluseviciute et al., 2024). In fact, since 2024, JASPAR has incorporated a TFBS extraction tool to retrieve predicted TFBS from a genomic region of interest, making it considerably easier to identify CREs to utilize in promoter design. Other key resources for plant TF-CRE interactions include PlantTFDB and PlantRegMap, which provide TF annotations, predicted binding motifs, and regulatory network predictions, and ConnecTF, which curates experimentally validated TF–target interactions (Brooks et al., 2021; Jin et al., 2017; Tian et al., 2020). Standardized classification of TFs would enable easier recognition of patterns within TF-CRE interactions across TF families, strengthening the prediction of potential interactions that are identified in experimentally gathered data.

## 4 TF-CRE interactions

**Figure 2:**
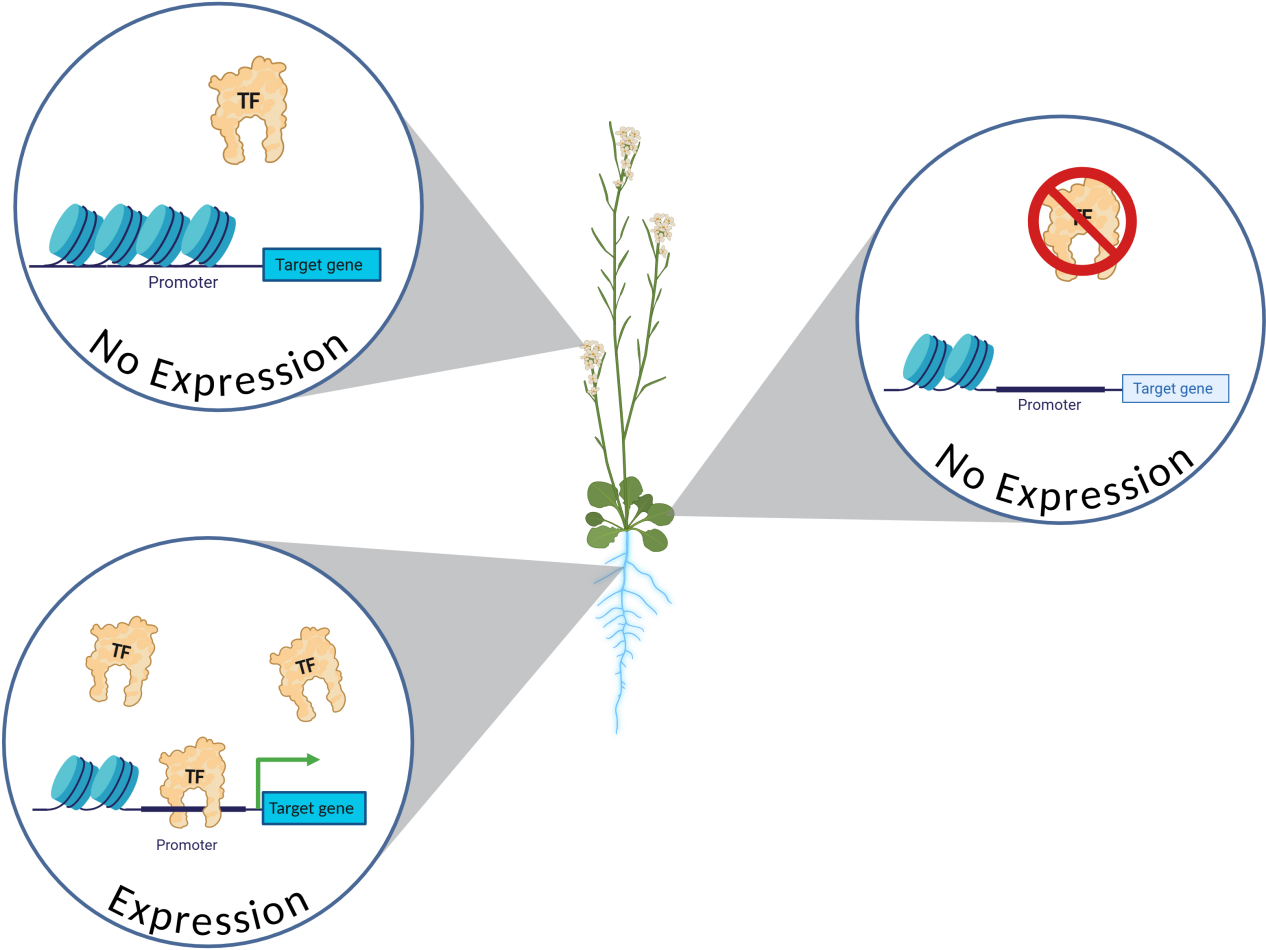
Example of TF-promoter interactions necessary for expression in a genomic context. In order for a gene to be expressed, both the TF and promoter need to be present and accessible in a given cell. Here, it is illustrated that the TF is present in the flowers of the plant, but the promoter is inaccessible due to the chromatin state. In the leaves, the promoter is accessible, but the TF is not present. In the root, the TF is present, the promoter contains a binding site for the TF, and the promoter is physically accessible, allowing TF occupancy. Light blue coloring of tissue represents activation of target gene expression. Created in https://BioRender.com.

The classification of TFs into families provides the first step towards a systematic analysis of TF function. Another essential layer of information is the identification of the consensus sequences bound by a TF or a TF family. The presence of a promoter motif alone does not guarantee activation of a gene, as transcriptional activity depends heavily on the chromatin accessibility of the motif and TF-binding affinity. Although a variety of approaches exist to determine the binding affinity of a TF to a DNA sequence, DNA Affinity Purification sequencing (DAP-seq) has made a significant contribution to the systematic assignment of TFs and their corresponding TFBS. DAP-seq is a prominent technique for investigating all possible interactions that exist between TFs and CREs based on binding affinity alone, precluding chromatin or DNA accessibility (Hutin et al., 2023). The study that introduced DAP-seq to the community did so by generating genome-wide TF binding profiles for hundreds of Arabidopsis TFs (O’Malley et al., 2016). These datasets have since been widely re-used and expanded as motif libraries for recognition of CREs within promoters and CRE enrichment analyses (Lavrekha et al., 2022). In order to elicit transcription of a gene, three conditions need to be true: 1) The TFBS exists within the promoter of the target gene, 2) the TFBS is accessible, and 3) its corresponding TF must be present in the cell or tissue of interest (**Figure 2**). DAP-seq allows for the elucidation of DNA binding landscapes for native TFs, which sets the stage for the identification of novel TFBSs and provides a much stronger foundation for motif-based promoter design than relying on compiling identified motifs from various isolated experiments.

TF activity is not only determined by its availability in a given cell, but also by its physical accessibility to its binding site within a genomic context (**Figure 2**). Epigenetic modifications like DNA methylation, histone modifications, chromatin accessibility, and nucleosome positioning can all affect the ability of TFs to interact with their TFBSs in a genomic context. In recent years as sequencing technologies have progressed, it has become easier to identify and map these modifications and describe chromatin accessibility at a whole-genome scale with techniques like Chromatin immunoprecipitation sequencing (ChIP-seq), micrococcal nuclease sequencing (MNase-seq), bisulfite sequencing (BS-seq), assay for transposase-accessible chromatin sequencing (ATAC-seq), single-molecule long-read accessible chromatin mapping sequencing (SMAC-seq), CUT&RUN, CUT&Tag, DNase-seq, and FAIRE-seq (Buenrostro et al., 2013; Frommer et al., 1992; Haring et al., 2007; Schones et al., 2008; Shipony et al., 2020; Skene and Henikoff, 2017; Kaya-Okur et al., 2019; Song and Crawford, 2010; Giresi et al., 2007). However, the data collection of genome-wide epigenetic modifications under various environmental and developmental contexts in plants is still in its infancy, with prohibitive costs and time-consuming protocols continuing to serve as obstacles for many plant researchers today. Still, the repository of plant species-specific epigenetic landscapes is steadily growing, with databases like AraENCODE, PlantCADB, PCSD, and PlantEMS serving as essential resources for identifying chromatin accessible regions within plants (Dao et al., 2025; Ding et al., 2023b; Liu et al., 2018; Wang et al., 2023). Considering the epigenetic context of TF-TFBS interactions is key part of determining the circumstances under which a promoter is active.

The binding of TFs to TFBS within promoters is inherently a dynamic process, with TFs constantly binding and dissociating from the DNA (Lu and Lionnet, 2021). Association and dissociation of TFs occur within seconds, allowing for rapid responsiveness to ever-changing cellular conditions. In yeast, it has been observed that free-floating TFs can “exchange” with molecules already bound to DNA, which increases the length of transcriptional bursting, a term that is used to describe the way in which RNA is transcribed (i.e., in bursts of activity with intermediate periods of no transcription (silence)) (Hebenstreit and Karmakar, 2024; Pomp et al., 2024). While this phenomenon has not been studied in plants, these mechanisms are likely the same in plants as in yeast due to the structural conservation of TF–DNA binding domains. This dynamic turnover is particularly important for promoters integrating multiple inputs, as it allows competitive and cooperative interactions among TFs to rapidly tune expression in response to environmental or developmental cues (de Jonge et al., 2020).

Yet another layer of complexity within TF-TFBS interactions lies in the cooperativity of particular TFBSs through interactions between their associated TFs or co-factors recruited by those TFs. Multiple motifs within a promoter can function synergistically through cooperative binding of their TFs to enhance expression or antagonistically via TF competitive binding or recruitment of repressors to restrict activity and by keeping adjacent chromatin in an accessible state (Sawant et al., 2005). This combinatorial behavior allows plants to integrate multiple exogenous and endogenous signals to produce expression patterns with specificity, employing natural genetic networks to achieve discrete spatiotemporal expression landscapes. In promoter engineering, these combinatorial interactions pose both a challenge and an opportunity. Though potentially useful in synthetic promoter design, the complexity of combinatorial effects of motifs can further complicate the process of identifying activity landscapes of CREs. On the other hand, when arranged correctly, motifs can be used to build promoters that act as Boolean logic gates, allowing for complex activation or repression of transcription that can be controlled by researchers.

## 5 Promoter architecture and grammar

Given the context-dependent nature of TF-TFBS interactions, elucidating the rules governing promoter architecture and grammar is essential for rational design of synthetic promoters with predictable transcriptional outputs. Promoter components are generally categorized into core, proximal, and distal regions, each contributing to transcriptional control (Brooks et al., 2023) (**Figure 1B**). Core promoters, also referred to as minimal promoters, define basal transcription, while motifs in the proximal-distal region confer regulatory responsiveness. Additionally, long-range regulatory elements found kilobases upstream or downstream of the TSS that function similarly to motifs found in promoter regions can enhance or suppress expression, adding additional layer of transcriptional control. The core promoter, defined as the region nearest to the transcription start site, contains motifs such as the TATA box (bound by TBP), CCAAT box (bound by CCAAT-binding factor/NF-Y heterotrimers (NF-YA/B/C) that recruit additional co-regulators), and the initiator element (Inr) (recognized by TFIID subunits) that support basal transcription initiation (Yamamoto et al., 2007) (**Figure 1C**). In plants, core promoters can also contain a Y-patch motif, a direction-sensitive pyrimidine-rich DNA sequence, though its specific DNA-binding proteins remain poorly characterized (Yamamoto et al., 2007; Brooks et al., 2023). Together, these motifs establish the basic framework necessary for basal transcriptional activity upon which additional regulatory layers are built.

It is important to note, however, that not all plant promoters contain a canonical TATA box. Less than 40% of promoters in *Arabidopsis thaliana* contain a TATA box (Molina and Grotewold, 2005). Some core promoters, including a number of those driving expression of photosynthesis-related genes, are classified as TATA-less. These promoters often rely on alternative motifs such as downstream promoter elements (DPEs) to recruit transcriptional machinery (Brooks et al., 2023). While generally less robust than their TATA-containing counterparts, TATA-less promoters expand the diversity of core promoter parts available to researchers for promoter engineering purposes.

A critical advancement in understanding plant core promoters came from research investigating the CaMV *35S* promoter. Landmark studies demonstrated that the 35S promoter is constitutive and modular, containing a downstream region that is sufficient for basal transcription initiation and an upstream region that greatly boosts expression (Odell et al., 1985). Subsequent dissection of this promoter revealed that individual domains or motifs contribute distinct expression patterns depending on developmental stage or tissue type, and that the displayed novel patterns can emerge from synergistic interactions among domains (Benfey and Chua, 1990). These investigations established the most widely known and commonly utilized core promoter within plant research to date, a minimal promoter sequence from the CaMV *35S* promoter spanning from -46 to +1 relative to the TSS (Odell et al., 1985; Benfey and Chua, 1990). Further studies utilized domain swapping of elements within the *35S* promoter to engineer diversified promoter sequences to avoid repetitive sequence elements that may induce transgene inactivation *in vivo* (Amack et al., 2022; Bhullar et al., 2003, 2007).

The proximal promoter, located directly upstream of the core region, is enriched in TF-binding sites that determine gene responsiveness to developmental and environmental stimuli. This region is where the bulk of CREs for regulators are found, enabling binding of TFs that can trigger or inhibit recruitment of the transcriptional machinery (**Figure 1C**). The bp range of the proximal promoter does not have a universal definition and may be species-specific, but is generally defined as up to 2kb as demonstrated in Arabidopsis as -1000 bp to +500 bp or in peach as -500bp to +200 bp of the TSS (Deng et al., 2023; Ksouri et al., 2021). In Arabidopsis, 86% of TFBSs are found -1000bp to +200bp of the TSS, with a bell-shaped peak at -50bp, though this positional preference may slightly change based on TF family (Yu et al., 2016). Thus, proximal promoters often contain many CREs in various arrangements, creating *cis*-regulatory modules (CRMs). These CRMs can overlap, which complicates attempts to deconvolute promoter structure and elucidate CRE or CRM function. The types of CREs and CRMs found in the proximal promoter region span from developmental and tissue-specific to hormone and environmentally responsive elements, such as CREs that mediate response to light, physical stress, or biotic and abiotic factors. Some well-known motifs that fit these categories include G-box (stress-responsive), W-box (pathogen-responsive), ABA-responsive element (ABRE), auxin-responsive element (AuxRE), and EIN3/EIL-binding site (EBS) (Ali and Kim, 2019; Birkenbihl et al., 2017; Fernandez-Moreno et al., 2025). Additionally, expanding the search window for CRE discovery beyond promoters can reveal additional CREs. For example, recent study in maize searched a broad window spanning −10 kb to +10 kb relative to the TSS to identify putative CREs (Qiu et al., 2025). Understanding the interactions and combinatorial effects of these CREs within CRMs is crucial for deciphering how promoters modulate expression in desired tissues or under specific environmental conditions.

Although less extensively studied than their animal counterparts, distal insulator or enhancer regions also contribute to transcriptional regulation in plants. Distal elements are located kilobases upstream or downstream of the proximal and core promoters (**Figure 1B**). TF occupancy studies in Arabidopsis have revealed that there are many distal sequences that function as distal CREs (DREs) and participate in long-range regulatory interactions through DNA looping (Deng et al., 2023). This, of course, relies on the chromatin accessibility of those regions, which means that distal elements may affect different sets of genes depending on the cell type. Genes may have any number of distal regulatory regions containing CREs as either enhancers, insulators, or silencers of transcription (**Figure 1C**). The existence of these distal elements expands the complexity of transcriptional regulation and highlights the importance of considering higher-order chromatin context in the design of regulatory DNA modules.

Understanding the rules that govern promoter function is essential for designing synthetic promoters capable of precise transcriptional control. Promoter activity in plants is not dictated solely by the presence of individual CREs. Rather, activity arises as a result of the arrangement, spacing, and combinatorial interactions of these motifs within a promoter sequence, a concept often described as promoter “grammar” (Weingarten-Gabbay and Segal, 2014) (**Figure 1C**). This concept of promoter grammar encompasses the spatial organization and number of motifs as well as their relative distances from the TSS and from each other, which determine the interactions between the TFs that bind them. Classical work has long noted that motif copy number, spacing, and orientation may affect promoter strength, while more recent studies demonstrate that even subtle changes, such as helical phasing (i.e. the TF binding face on the 10.5 bp turn in the DNA helix), can have pronounced effects on gene expression levels (Georgakopoulos-Soares et al., 2023; Huang et al., 2012; Rushton et al., 2002) (**Figure 1C**). Effects of helical phasing on expression in particular highlights the importance of considering interactions between linear sequence features with three-dimensional DNA architecture when studying expression dynamics of novel promoters.

An increase in the copy number of motifs in a promoter region has been well-known to correlate with an increase in transcriptional activity (Rushton et al., 2002). The rationale is that additional motif copies provide more binding sites for TFs, thus raising the probability of TF binding and, in some cases, enabling the cooperative or simultaneous binding of multiple TFs (Brophy et al., 2022; Fernandez-Moreno et al., 2024). Increasing the number of motifs can lead to stronger or more stable transcriptional activation by encouraging protein-protein interactions or keeping chromatin accessible, especially when the respective TF is abundantly present in the cell.

As mentioned earlier, one critical aspect of promoter grammar is motif spacing. Experimental studies in plants and other eukaryotes have demonstrated that even small changes in the distance between binding sites can significantly alter transcriptional output (Bhadouriya et al., 2021; Rushton et al., 2002). Closely spaced motifs may facilitate cooperative binding of TFs, enhancing transcriptional activation, whereas motifs placed too far apart may fail to interact synergistically. On the other hand, motifs that are placed too close to one another may introduce the issue of steric hindrance when it comes to their respective TFs binding and thus cause repression of transcription. Synthetic promoter libraries in Arabidopsis have been used to systematically vary spacing between motifs, revealing nonlinear effects on gene expression and highlighting the importance of precise spatial arrangement in promoter design (Bhadouriya et al., 2021). Additionally, TFs may interact preferentially with motifs in specific orientations (Zhu et al., 2018). Studies have shown that reversing the orientation of a motif or changing its position relative to other motifs can modulate transcriptional activity, sometimes producing unexpected synergistic or antagonistic effects (Xie et al., 2025).

Another important consideration is the sequence context surrounding promoters and promoter motifs. The activity resulting from the presence of a motif often depends on its position relative to the transcription start site, the presence of neighboring motifs, chromatin accessibility, and the endogenous TF landscape within the cell or tissue (Jensen and Galburt, 2021; Sijacic et al., 2018). Particularly within the context of the latter, TFs exhibit varying degrees of specificity and may preferentially bind to different sites based on cell type and epigenetic state of the chromatin, resulting in a gradient of transcriptional output rather than a binary on/off state (Yaschenko et al., 2022). Additionally, motif arrangement can greatly impact transcriptional activity, as motifs can act additively, synergistically or antagonistically in collaboration with other motifs present, with most motifs interacting in a ‘more-than-additive’ manner (Jensen and Galburt, 2021). As previously discussed, enhancers, nucleosome positioning, and local DNA methylation can modulate TF binding and consequently promoter activity (Chen et al., 2024). Thus, the same motif arrangement can yield different outcomes depending on cellular context, emphasizing the need to integrate promoter architecture with knowledge of TF expression and chromatin state when designing synthetic promoters.

Understanding promoter grammar and architecture facilitates the design of modular, context-adaptable synthetic promoters. While the complexity of native promoter architecture continues to be an obstacle in achieving tunable gene expression in plants, the modularity of promoter elements provides a foundation for promoter engineering (Yaschenko et al., 2022). Deciphering the rules of promoter grammar can enable researchers to rationally design synthetic promoters that produce precise, predictable expression patterns and levels in both foundational and applied plant research.

## 6 Synthetic promoter design in plants

A synthetic promoter is defined as any promoter artificially designed by man, assembling existing CREs in combinations that are not found in nature or even creating new CREs. These types of promoters have been traditionally leveraged to drive expression in a spatiotemporal-specific manner to create novel expression profiles for genes of interest. Synthetic promoters have also been utilized to further understand general promoter structure and motif function, as many early synthetic promoters were built by combining pieces of native promoters to produce a novel promoter that is an amalgamation of motifs that respond to various signals (Khan et al., 2023). This type of synthetic promoter building is often referred to as promoter shuffling, where CREs or CRMs from existing promoters get mixed around for the purpose of achieving more desirable expression patterns (Khan et al., 2023). In more recent years, it has become popular to utilize computational methods to design synthetic promoters from promoter elements with known expression patterns, which, in a way, is just a more elaborate and sophisticated form of promoter shuffling (Yasmeen et al., 2023).

**Figure 3:**
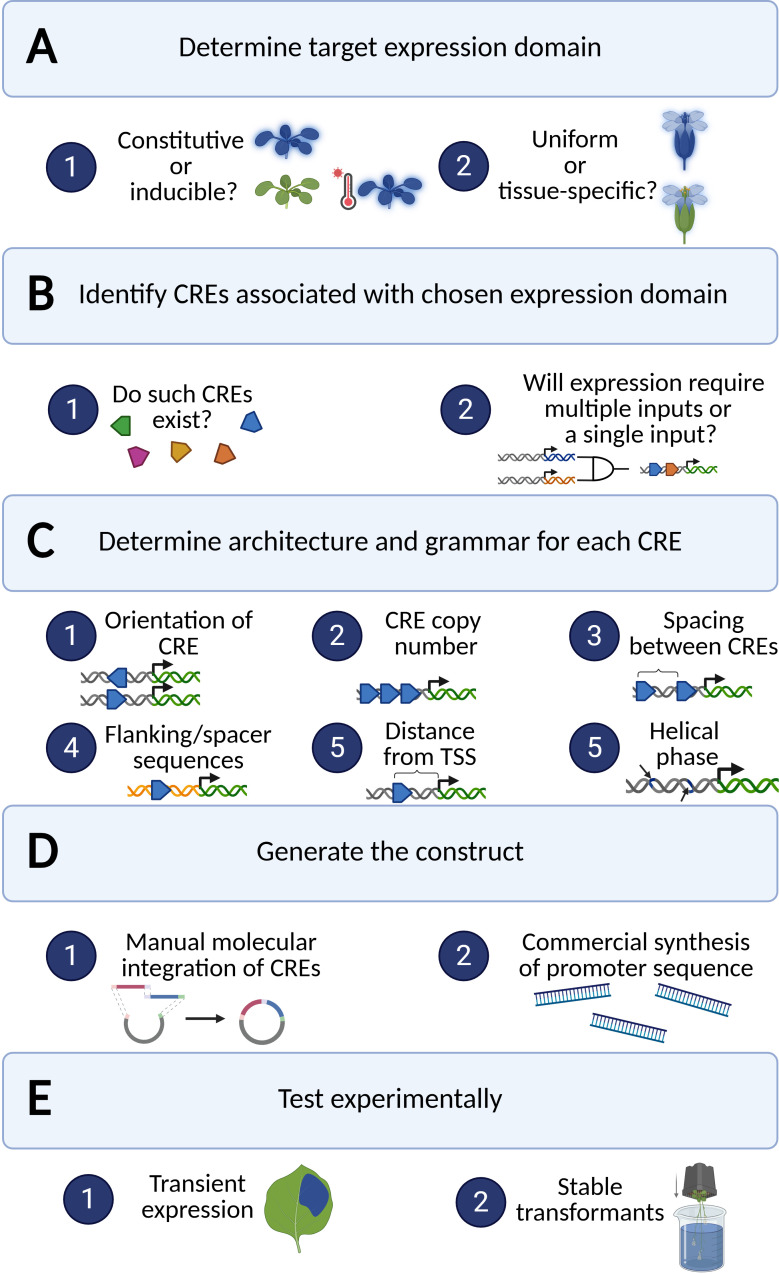
Workflow of designing a synthetic promoter. Each of the panels, **(A–E)**, represents a step in the process of designing synthetic promoters, with the dark blue circles that contain numbers representing questions to ask or options to consider. Created in https://BioRender.com.

The design and assembly of a synthetic promoter begin with identifying CREs that can be harnessed to achieve desired promoter behavior, whether it be constitutive, inducible, or tissue-specific expression (**Figures 3A, B**). Synthetic promoters can range from containing single CREs to tandem arrays of multiple homotypic or heterotypic motifs. These motifs can associate with one or more TF families, with more intricate synthetic promoter designs combining motifs from different regulatory pathways into one promoter sequence to enable transcriptional response to multiple independent signals (Li et al., 2023). This combinatorial approach allows for the creation of synthetic promoters with novel expression patterns or stimulus responsiveness. Early synthetic promoter experiments have shown that multimerization of these motifs upstream of a *35S* minimal promoter confers stimulus-specific expression, with an increase in expression as copy number increases (Rushton et al., 2002). The Rushton et al. study was also an early indicator of the importance of motif arrangement and spacing, aspects of promoter grammar, as well as dependency of expression on flanking sequences (Rushton et al., 2002). Modern studies have reinforced these findings, highlighting the importance of considering promoter architecture and grammar when designing synthetic promoters (Jameel et al., 2020; Jores et al., 2021; Smaczniak et al., 2017).

As noted earlier, interactions between motifs can produce synergistic or antagonistic effects on transcription in a non-linear manner depending on sequence context, motif identity, and TF availability. This is especially important to consider when relying on TFs that act cooperatively, requiring all TF partners to bind to their respective TFBS to activate transcription, as exemplified with the interaction of CONSTANS (CO) and NUCLEAR FACTOR Y (NF-Y) in the regulation of *FLOWERING LOCUS T* (*FT*) expression in Arabidopsis (NF-Y binds to a distal *CCAAT* element while CO binds at proximal “CORE” elements to induce *FT* expression) (Siriwardana et al., 2016). Understanding how types of motifs, repetitions of each motif, and arrangement of motifs within a promoter affect promoter function is essential to producing synthetic promoters that behave predictably (**Figure 3C**). Fortunately, there are resources available that can aid in pinpointing favorable motifs for a given application. Extensive motif catalogs, such as those compiled in PlantTFDB, PlantRegMap, and ConnecTF, document thousands of TFBSs across plant genomes, providing valuable resources for synthetic promoter construction (Brooks et al., 2021; Jin et al., 2017; Tian et al., 2020). Additionally, mapping of DNase I hypersensitive sites (DHS) has been conducted in plants to capture genome-wide landscapes of open chromatin to enable the identification of CREs across plant genomes, a goal that could also be accomplished using other, complementary methods that capture chromatin accessibility, such as ATAC-seq (Zhang et al., 2012). The JASPAR database further complements these plant-specific resources by offering a curated, open-access collection of TF binding profiles across multiple species, including plants (Rauluseviciute et al., 2024).

There are a number of ways to generate synthetic promoters depending on experimental set-up and resource availability (**Figure 3D**). Site-directed mutagenesis (SDM) allows for the integration of desired CREs into an otherwise CRE-depleted sequence or a native promoter in a transgene *in vitro* (Bachman, 2013), while targeted genome editing techniques can be utilized to edit CREs in native promoters *in vivo*, allowing for the study of gene regulation or promoter spatiotemporal activity without the need to introduce a transgene (Saeed et al., 2022). Depending on synthetic promoter length, promoters may be synthesized entirely by commercial DNA synthesis companies, either in whole or in parts, then assembled into transcriptional units via molecular assembly methods (Fernandez-Moreno et al., 2024; Sarrion-Perdigones et al., 2013) (**Figure 3D**). In this case, it is necessary to determine whether CREs or CRMs will be placed directly in tandem or if the incorporation of spacer sequences is necessary. These spacer sequences can either be regions located between CREs of a native promoter or can be entirely artificial sequences, specifically sequences with no known TFBSs. It is imperative to verify whether the final synthetic promoter sequence resulting from joining spacer sequences with CREs gives rise to unintentional TFBSs, as these can affect the ability of the promoter to behave as intended. Deliberate comparison of novel promoter sequences to existing databases of known TFBS, like JASPAR, is essential to mitigating this potential issue (Rauluseviciute et al., 2024).

Despite recent advancements in predictive modeling that will be expanded upon later in this review, experimental validation continues to be vital to evaluating the effectiveness of a given synthetic promoter design (**Figure 3E**). These newly constructed promoters are typically assessed through transient reporter assays or stable integration into the genome of model organisms like Arabidopsis. High-throughput techniques, like massively parallel reporter assays or STARR-seq approaches, have emerged to quantify activity of synthetic promoter libraries across various conditions and tissues (Tan et al., 2023). For example, Schaumburg et al. characterized more than 120 synthetic parts in Arabidopsis and more than 100 parts in sorghum protoplasts, utilizing dual luciferase assays expressing Firefly and Renilla synthetic reporters transiently (Schaumberg et al., 2016). Other studies have employed various reporters to conduct similar transient assays in order to rapidly characterize synthetic promoters (Cai et al., 2020). This type of empirical testing not only reveals whether the synthetic promoters function as expected but also begins to build a database of verified synthetic promoter activity levels that can later be utilized as a training set for computational models.

Thanks to the vast amount of time and energy researchers have put into building synthetic parts for various applications, the plant research community has developed a large repertoire of genetic parts with diverse functions that can be leveraged in designing synthetic promoters (Feike et al., 2019; Koo et al., 2025). Cai et al. developed a suite of minimal (core) synthetic promoters, named MinSyns, that can be utilized in plants, increasing the diversity of core promoters that can be employed in higher complexity regulation of expression involving multiple genes (Cai et al., 2020). This suite of promoters grants researchers flexibility in controlling target gene expression and aids in avoiding the reuse of the same sequences in a single transgene construct. Jores et al. analyzed massive self-transcribing active regulatory region sequencing (STARR-seq) libraries containing thousands of core promoters for Arabidopsis, maize, and sorghum, then leveraged key features identified in that analysis to build synthetic promoters for Arabidopsis and maize (Jores et al., 2021). As mentioned above, Schaumberg et al. also added over 200 novel synthetic parts to the repository of promoters available to the plant research community (Schaumberg et al., 2016). More recently, Zhou et al. developed and meticulously characterized a set of 15 full-length constitutively expressing synthetic promoters across multiple species (Arabidopsis, *Nicotiana benthamiana*, *Medicago truncatula*, and *Lactuca sativa*) (Zhou et al., 2023). The continued development of synthetic promoters and employment of either high-throughput or manual validation of promoter activity have greatly advanced our understanding of promoter design. These resources broaden the design space for synthetic genetic circuits and support the construction of larger, more complex networks without reusing synthetic parts, which could risk transgene silencing.

## 7 Applications of synthetic promoters

Synthetic promoters can be used as a tool to study expression domains of CREs, create biosensors for the research community, and to enable combinatorial control of gene expression for complex trait engineering using genetic logic gates. The examples reviewed in this section are not comprehensive but rather highlight classical as well as more recent applications of synthetic promoters.

### 7.1 Functional genomics

One of the earliest and most popular applications of synthetic promoters has been their use in functional genomics to drive constitutive tissue-specific or condition-specific expression of target or reporter genes (Ulmasov et al., 1997; Rushton et al., 2002). Since natural promoters can behave in unexpected ways, synthetic sequences composed of modular CREs upstream of minimal promoters have proven advantageous in generating more precise patterns of expression (Brooks et al., 2023). However, some studies have had success with modifying natural promoters to create synthetic ones, either through mutations, deletions, or chimeric fusions of multiple natural promoter sequences or elements (Efremova et al., 2020; Kumari et al., 2024; Sherpa and Dey, 2025). Two recent papers reported the creation of copper-inducible systems in *Nicotiana benthamiana* by utilizing four copies of the *copper-binding site (CBS)* motif fused upstream of various minimum promoters to create synthetic promoters that a copper responsive TF, CUP2, can bind to only in the presence of copper (**Table 1**) (Garcia-Perez et al., 2022; Chiang et al., 2024). In one of these studies, the copper-inducible system outperformed other inducible systems that were tested, specifically systems that rely on β-estradiol, dexamethasone, or doxycycline (Chiang et al., 2024). Another study leveraged promoters from chickweed *ANTIMICROBIAL PEPTIDE1* (*AMP1*) and *ANTIMICROBIAL PEPTIDE2* (*AMP2*) genes to create a constitutive chimeric promoter that expresses higher than *pro-SmAMP2* and similarly to *pro-SmAMP1* in transient assays, exhibiting a diversified promoter sequence that can achieve levels of activity similar to a natural promoter (**Table 1**) (Efremova et al., 2020). Additionally, the study was able to identify proline-inducible motifs from these natural promoters that when excluded from *pro-SmAMP2*, led to high constitutive expression profiles. Though this may not seem like a very significant finding, it is important to remember that generating promoters with diverse sequences that are able to constitutively express target genes is one of the goals of synthetic biology research, as these diversified promoter libraries are essential to downstream generation of genetic logic gates with non-overlapping inputs. To this end, natural promoters from plant pararetroviruses can also be leveraged to create synthetic promoters that constitutively express in plants, as seen with the creation of *35S* promoter variants (Ali and Waliullah, 2021; Amack et al., 2022). *MSD3* is an example of a synthetic promoter that is an amalgamation of motifs from various monocot and dicot pararetroviral-based promoters that is able to drive expression at similar levels to the *35S* gold-standard constitutive promoter in rice, pearl millet, and tobacco plants (**Table 1**) (Kumari et al., 2024). Another synthetic promoter that was recently developed in the same manner, *MFH17*, contains fused elements from *Mirabilis mosaic virus* (*MMV*), *Figwort mosaic virus* (*FMV*), and *Horseradish latent virus* (*HRLV*) derived promoters and is also able to drive high expression in both dicots and monocots (**Table 1**) (Sherpa and Dey, 2025). These promoters represent valuable additions to the growing library of constitutive promoters available for use in plant biotechnology. Their effectiveness highlights the significance of a combinatorial approach, specifically by exploring and integrating promoter elements from diverse species to develop highly efficient, strongly expressing synthetic promoters.

**Table 1:** Compilation of key synthetic promoters and their applications.

Type of promoter	Promoter name	Application
Copper-inducible	*4xCBS* (Garcia-Perez et al., 2022; Chiang et al., 2024)	Responds to the presence of copper in *Nicotiana benthamiana*
Constitutive	*pro-SmAMP1 + proSmAMP2* chimeric promoter (Efremova et al., 2020)	Can be utilized as a stronger constitutive promoter than the canonical *CaMV35S* promoter commonly employed in plants
Constitutive	*MSD3* (Kumari et al., 2024)	Constitutively expresses higher than *CaMV35S* in both monocots and dicots
Constitutive	*MFH17* (Sherpa and Dey, 2025)	Constitutively expresses higher than *CaMV35S* in both monocots and dicots; created to diversify constitutive promoter library
Green-tissue- specific	*BiGSSP2,3,6,7* (Bai et al., 2020)	Enable bi-directional expression of reporters in green tissues of rice
Green-tissue- specific	*Syn3* (Yang et al., 2024)	Enables constitutive expression of target genes in green tissues of poplar
Drought-inducible and green-tissue specific	*Syn3-10b-1* (Yang et al., 2024)	Responds to changes in abiotic stress, particularly drought, in green tissues of poplar
Drought-inducible and root-tissue specific	*SynP16* (Jameel et al., 2020)	Responds to changes in abiotic stress, particularly drought, in the roots of soybean and Arabidopsis
Hormone biosensor	*DR5; DR5v2* (Ulmasov et al., 1997; Liao et al., 2015)	Detect levels of auxin in plant tissues
Hormone biosensor	*5xEBS; 10×2EBS-S10; EBSn* (Stepanova, 2001; Stepanova et al., 2007; Fernandez-Moreno et al., 2024, 2025)	Detect levels of ethylene in plant tissues
Hormone biosensor	*6xABRE* (Wu et al., 2018)	Detects levels of ABA in plant tissues
Hormone biosensor	*TCS; TCSn; TCSv2 (*Müller and Sheen, 2008; Steiner et al., 2020; Zürcher et al., 2013)	Detect levels of cytokinin in plant tissues
Hormone biosensor	*AuxRE* + *2xCKRE* (Li et al., 2023)	Responds to the presence of both auxin and cytokinin acid in plant tissue; helpful for studying hormone crosstalk
Hormone biosensor	*SJ-609* (Li et al., 2023)	Responds to the presence of both salicylic acid and jasmonic acid in plant tissue; can also be utilized as a pathogen sensor
Pathogen sensor	*4xS-Box* (Persad-Russell et al., 2022)	When used in conjunction with a *Neurospora crassa* transcriptional activator variant QF2, can detect *Clavibacter michiganensis* subsp*. nebraskensis* presence in potato
Salt-inducible	*PS* (Bhadouriya et al., 2024)	Responds to changes in abiotic stress, particularly salt levels

In addition to constitutive promoters, researchers have also explored creating tissue-specific promoters for various purposes. Four synthetic promoters created through informed fusion of regulatory sequences derived from natural promoters, *BiGSSP2, BiGSSP3, BiGSSP6*, and *BiGSSP7*, were shown to induce bi-directional (i.e. can initiate transcription in either orientation) expression of reporters in rice (**Table 1**) (Bai et al., 2020). These promoters function specifically in green tissues, including leaf, sheath, panicle, and stem, and represent a promising example of how synthetic promoter design can provide agronomically relevant tissue specificity with direct potential for crop improvement (Bai et al., 2020). The capacity to restrict gene expression to photosynthetically active tissues is particularly valuable for metabolic engineering applications, where minimizing ectopic expression reduces unintended fitness costs while still achieving strong expression in target organs.

Identifying tissue-specific promoters with inducible functionality can provide a mechanism for tunable spatiotemporal control of gene expression. Inducible promoter designs are highly advantageous when driving expression of genes with potentially detrimental effects, as constitutive expression could cause cytotoxicity and compromise plant health. Tissue-specific inducible promoters ensure that expression is activated only when necessary in a restricted tissue domain. A study by Yang et al. (2024) exemplifies this approach by designing and testing a drought-inducible, green-tissue-specific promoter in poplar (**Table 1**) (Yang et al., 2024). Their methodology involved identifying relevant motifs from genes upregulated in leaf palisade and vascular tissues under water-deficit stress. This resulted in the creation of two synthetic promoters: a constitutive synthetic promoter *Syn3*, created by concatenating four repeats of a conserved 20 bp motif derived from promoters of green-tissue-specific genes differentially expressed under drought conditions, that preferentially expresses in green tissue, and a second promoter, *Syn3-10b-1*, created by concatenating the 5’ region of the 20 bp motif utilized in *Syn3*, that specifically drives gene expression only in green tissues under drought conditions in transgenic poplar (**Table 1**). Another study, through experimentation with copy number, orientation, and spacing of motifs, generated and characterized a suite of potential root-specific and drought-inducible synthetic promoters (Jameel et al., 2020). Root-specific promoters are of particular interest because root tissues are the primary site of water and nutrient uptake, and reprogramming their behavior under stress is critical for agricultural applications, such as enhancing crop resilience or adaptability. In the study by Jameel et al., one synthetic promoter in particular containing eleven concatenated *in-silico* identified CREs, *SynP16*, stood out in its specificity to root tissue in response to drought conditions in soybean and Arabidopsis (**Table 1**). The particular combination and arrangement of root-specific and drought-inducible elements in *SynP16*, though arising fortuitously in this case, illustrates how methodical researchers must be when constructing synthetic promoters if we hope to achieve highly controlled and context-dependent expression patterns.

### 7.2 Biosensors

The emergence of rational inducible synthetic promoter designs enabled researchers to monitor specific hormones, stresses, or metabolites in direct proportion to signal intensity through the use of synthetic promoter-driven reporters, commonly referred to as transcriptional biosensors. Auxin is arguably the most intensively studied plant hormone in terms of how it is spatially distribution and developmental patterning, and synthetic promoters were pivotal to that progress (**Table 1**) (Ulmasov et al., 1997; Liao et al., 2015). The canonical *DR5* promoter, constructed from multimerized *AUXIN RESPONSE ELEMENT* (*AuxRE*) motifs (i.e. TGTCTC) recognized by Auxin Response Factors (ARFs), has been used for decades to visualize auxin dynamics in roots, embryos, and shoots (Ulmasov et al., 1997). Switching to TGTCGG motifs and optimizing repeat motif orientation gave rise to the *DR5v2* promoter, resulting in increased sensitivity and refined cellular resolution (**Table 1**) (Liao et al., 2015). In more recent years, auxin responsive synthetic promoters have been leveraged to deconvolute auxin regulated transcription (Martin-Arevalillo et al., 2025). Preferential binding sites for auxin responsive factors (ARFs) in the form of *AuxRE* pair configurations were utilized in synthetic promoters to identify auxin-induced transcriptional responses at a single-cell level. The study found that different clades of ARFs prefer distinct binding sites, leading to spatial specificity of the *AuxRE* pairs *in planta*. This finding is yet another example illustrating how combinations of motifs can drive divergent expression patterns in plants.

Another useful hormone to create biosensors for is ethylene, due to its prominent role in plant growth and development (Dubois et al., 2018). The ethylene signaling pathway has been leveraged repeatedly to construct ethylene biosensors by generating synthetic promoters containing *EIN3*-binding sites (*EBS*) to drive fluorescent or histochemical reporters. In early efforts, Stepanova et al. developed a synthetic ethylene-responsive promoter using 5 copies of multimerized *EBS* motifs (**Table 1**) (Stepanova, 2001; Stepanova et al., 2007). This promoter was activated in the presence of ethylene but was limited in its sensitivity and expression uniformity across tissues. To address these restrictions, a new generation of ethylene biosensors was constructed by optimizing the sequence and arrangement of *EBS* motifs. One example is the *10×2EBS-S10* promoter, which contains ten tandem homotypic copies of a dual, everted *EBS* motif placed upstream of the minimal *35S(-46)* promoter (**Table 1**). This promoter displays moderate ethylene responsiveness in Arabidopsis seedlings, showing different expression domains than the original *5xEBS* promoter (Fernandez-Moreno et al., 2024). Building on this, an even more recent study reported the *EBSn* promoter, which is composed of ten heterotypic, natural, dual, everted *EIN3*-binding sites placed in tandem upstream of the same minimal *35S (-46)* promoter (**Table 1**) (Fernandez-Moreno et al., 2025). *EBSn* was found to outperform earlier ethylene reporters in terms of sensitivity and breadth of response, detecting changes in ethylene production in both seedlings and adult plants of Arabidopsis. In tomato fruits, *EBSn* reporters displayed ripening-related ethylene accumulation, showcasing the promoter’s utility for crop applications (Fernandez-Moreno et al., 2025).

These ethylene biosensors suggest two important design principles: 1) multiplicity of binding sites within a promoter amplifies signal but may suffer from sequence-stability and synthesis issues, and 2) promoters that concatenate heterotypic motifs associated with natural binding-site variation can outperform promoters with simple homotypic repeats by better matching the native TF recognition landscape. Both biosensors, driven by either *10x2EBS-S10* or *EBSn*, are state-of-the-art examples of how informed synthetic promoter design can result in robust tools for monitoring hormone signaling to aid fundamental plant research.

Motifs associated with other plant hormones have also been utilized to construct synthetic promoters for hormone biosensors. The *6xABRE* synthetic promoter harboring six tandem *ABRE* motifs upstream of a minimal promoter is a widely used ABA-responsive promoter that reveals spatiotemporal dynamics of ABA signaling (**Table 1**) (Wu et al., 2018). *TCS* and *TCSn* are the classic cytokinin-responsive synthetic promoters, built from concatenated type-B Arabidopsis response regulators (ARR) binding sites (**Table 1**) (Müller and Sheen, 2008). *TCSn* in particular offers improved sensitivity and has been leveraged as a template for the creation of other cytokinin-responsive promoters, such as *TCSv2*, which alters the orientation of the type-B ARR binding sites utilized in *TCSn* (**Table 1**) (Steiner et al., 2020; Zürcher et al., 2013). In more recent years, dual hormone-responsive promoters responsive to either auxin or cytokinin were created by combining CREs from both pathways (*AuxRE* for auxin and *2xCKRE* for cytokinin) into one promoter, enabling the study of hormone crosstalk and the design of conditional control circuits (Li et al., 2023). Synthetic promoters responsive to salicylic acid (SA) and jasmonic acid (JA) were created in a similar manner, given that these two hormones play a large role in plant defense against pathogens and thus could be extremely useful in both sensing pathogen presence and hormone levels (Li et al., 2023). The most promising synthetic promoter, *SJ-609*, was constructed by inserting a JA-responsive CRE into an SA-responsive promoter and conferred higher expression in response to either hormone, as well as pathogen presence, in tobacco, Arabidopsis, tomato, and cotton than either of the native JA or SA responsive promoters tested (**Table 1**) (Li et al., 2023). This study not only directly adds to the repertoire of inducible hormone biosensors but also showcases a novel design strategy for synthetic promoters that can respond to multiple alternative inputs simultaneously.

Another study developed pathogen-sensing constructs in potato using synthetic promoters built from S-box elements, motifs that are responsive to fungal infection, coupled to a transcription-factor system (Q-system) (Persad-Russell et al., 2022). Utilizing the *4×S-Box* synthetic promoter to drive a Q-system transcriptional activator variant QF2, which then binds to its TFBS upstream of an *mEmerald GFP* reporter gene, yielded approximately a six-fold signal amplification upon infection with *Clavibacter michiganensis* subsp*. nebraskensis* (CMN), demonstrating enhanced sensitivity for pathogen detection in potato (**Table 1**). This promoter, and others like it, would allow for early detection of plant pathogen infection in real-world applications, and possibly even trigger targeted defense responses in the plant upon pathogen encounter.

Ability to respond to salt and osmotic stress has also been explored through the design of synthetic promoters. Bhadouriya et al. reported a synthetic salt-inducible promoter, named *PS*, composed of motifs extracted from native salt-responsive promoters and rationally arranged based on their original configuration within their respective promoters (**Table 1**) (Bhadouriya et al., 2024). The PS promoter was used to drive reporter expression in transient assays within *Nicotiana tabacum* and in stably transformed Arabidopsis lines, showing stronger and more sustained expression under salt/abiotic stress conditions than CaMV *35S*. The results demonstrate that synthetic promoter design can outperform common constitutive promoters in a stimulus-dependent manner, validating the *cis*-engineering approach for abiotic stress biosensors (Bhadouriya et al., 2024).

### 7.3 Logic-gate-based genetic circuits

**Figure 4:**
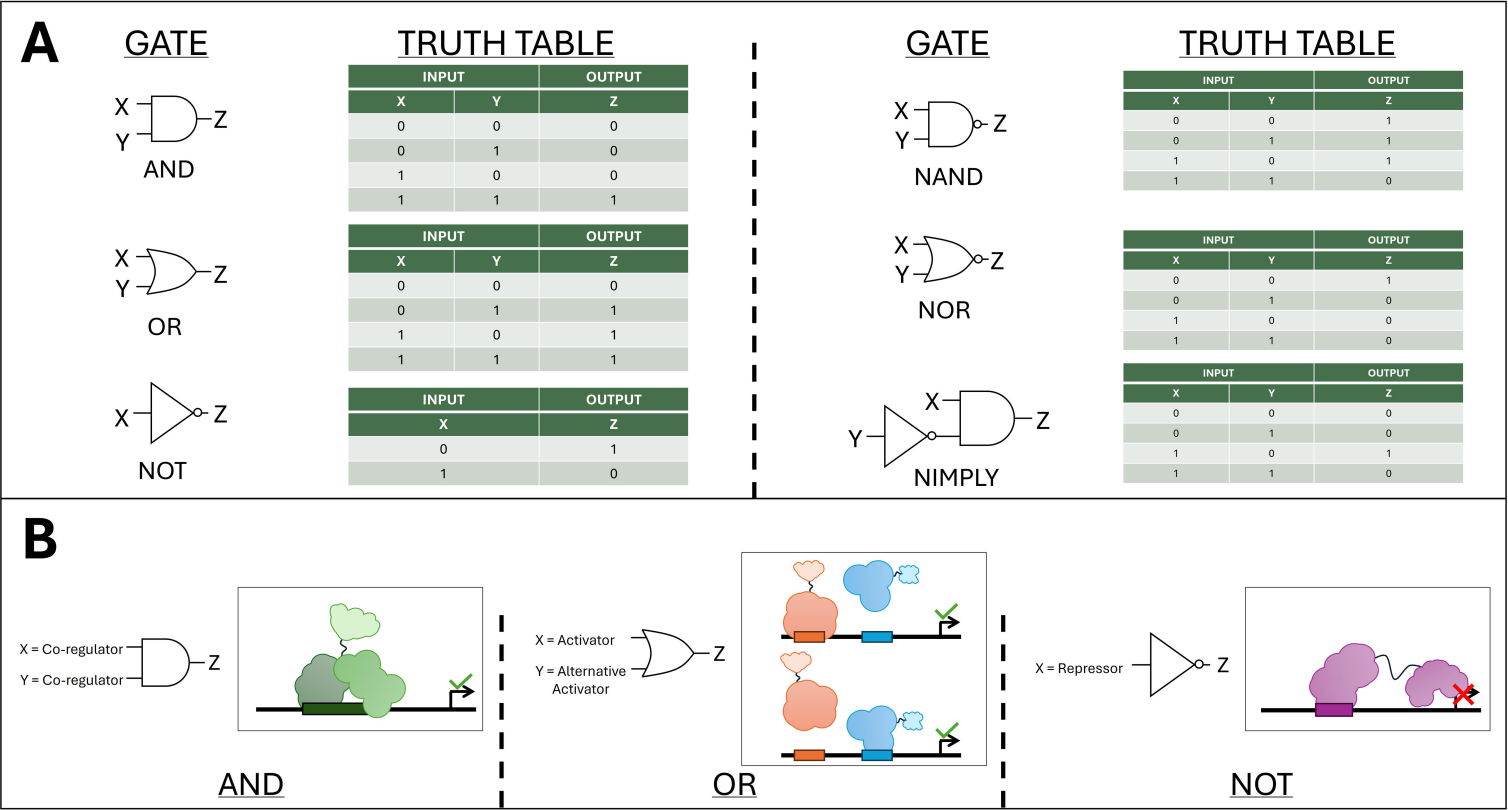
Illustration of logic gates. **(A)** Schematic of the AND, OR, NOT, NAND, NOR, and NIMPLY logic gates. X and Y represent the inputs to the gate, while Z represents the output. The truth tables depict all the different possible outputs given a set of inputs. The number 1 represents ‘TRUE’, while 0 represents ‘FALSE’. **(B)** Illustration of how the AND, OR, and NOT gates may be implemented to control transcription of a target gene (Z). Orange and blue represent transcriptional activators. Green TF shapes in the AND gate represent subunits of a heterodimeric TF, requiring both subunits to be present for activation of the target gene. Purple represents a repressor, which in the illustration is made up of a DNA-binding domain and a repression domain.

In recent years, synthetic promoters have begun to be utilized in the design of genetic logic gates in plants. Genetic logic gates borrow concepts from mathematics and Boolean logic to achieve conditional control of gene expression based on combinations of biological inputs such as transcription factors, signaling molecules, or environmental cues, allowing for complex and programmable transcriptional responses (Bradley et al., 2016; Andres et al., 2019). A logic gate takes in one or more binary inputs (i.e., that resolve to true (one) or false (zero)) and produces one binary output depending on the type of logic gate. In this framework, synthetic promoters allow for the integration of multiple CREs that respond to distinct upstream regulators. The resulting promoter output, either transcription activation or repression, functions analogously to Boolean logic operations such as AND, OR, and NOT (Miyamoto et al., 2013) (**Figure 4A**). At the transcriptional level, an AND gate may require two different TFs to bind simultaneously for activation, ensuring that expression only occurs when both signals are present (**Figure 4B**). Of course, given the nature of promoter activation by TFs, AND gates built in this manner are commonly ‘leaky’, as the presence of a single TF’s TFBS can often be sufficient for some level of expression. The idea is the TFs act cooperatively, significantly increasing expression when both TFBS are present (AkhavanAghdam et al., 2016). Conversely, OR gates can be designed by embedding multiple independent activator-binding sites, enabling expression when any one of several signals is detected (**Figure 4B**). Most current synthetic promoters are representative of the OR gate logic, as it is more difficult to identify motifs that can be combined to produce the AND logic described above. Similarly, NOT gates can be implemented through integrating repressor-binding motifs in the promoter of the output gene and expressing synthetic transcriptional repressors that block transcription in the presence of a specific input (**Figure 4B**). Such promoter-based logic designs enable precise, context-dependent expression of genes, allowing plants to make finely tuned transcriptional “decisions” in response to complex environmental or developmental conditions.

**Figure 5:**
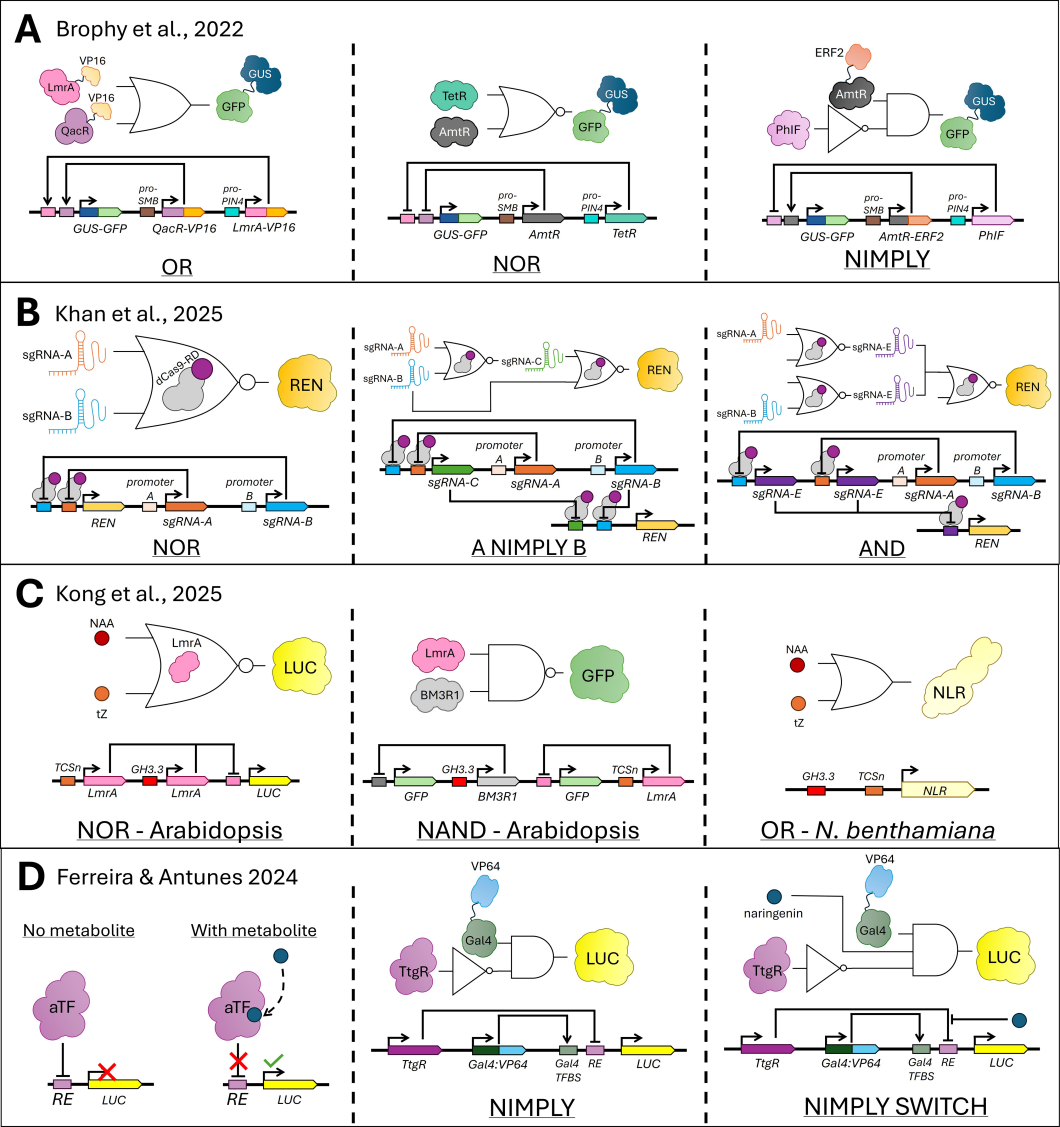
Implementation of genetic circuits in transcriptional regulation. Schematics of various genetic circuits created from logic gates using transcriptional activators, repressors, and inducers to control expression of a target gene. Rectangles represent TFBSs, while rectangles with a triangular side represent genes. Circles represent inducers. **(A)** Three gates implemented in Arabidopsis roots by Brophy et al., 2022 to control expression of a *GUS-GFP* reporter gene. The inputs for each gate are driven by either the *SOMBRERO (SMB)* promoter or the *PIN-FORMED4* (*PIN4*) promoter, each of which is specific to a subset of root cells. **(B)** Three gates implemented in Arabidopsis protoplasts by Khan et al., 2025 to control expression of a *Renilla luciferase* (*REN)* reporter gene. Left panel illustrates the general structure of the NOR gate, with the sgRNA targeting dCas9 fused to a repression domain (RD) to the promoter of *REN*. The middle panel depicts one of the NIMPLY gates, A NIMPLY B, while the right panel depicts the AND gate created by combining two layers of NOR gates, with various engineered sgRNAs serving as the inputs and *REN* expression serving as the output. **(C)** Three gates implemented in either Arabidopsis protoplasts (left), stable transgenic Arabidopsis lines (middle), or *N. benthamiana* leaves (right) by Kong et al., 2025. The Arabidopsis-implemented gates rely on repressors that are inducible by either NAA or tZ to control expression of either firefly *Luciferase* (*LUC*) or *GFP* reporter genes. The *N. benthamiana-*implemented OR gate utilizes NAA- and tZ-responsive promoter elements to modulate expression of an *NLR* gene that elicits a hypersensitive response in *N. benthamiana* leaves, leading to cell death. **(D)** Three gates implemented in Arabidopsis protoplasts by Ferreira and Antunes, 2024 to control expression of the *LUC* reporter gene. Left panel illustrates the function of the bacterial repressor allosteric TF (aTF) utilized to control expression of a *LUC* target gene, with the blue circle depicting the phenylpropanoid metabolite associated with any given aTF. The right two panels illustrate a NIMPLY gate either with (right) or without (middle) naringenin, a phenylpropanoid metabolite, as a third input into the gate, allowing for disruption of aTF-associated repression of the target gene, *LUC*.

A landmark study of synthetic promoter utility in plants, provided by Brophy et al., demonstrated how logic circuits could be constructed from synthetic promoters to reprogram root architecture in Arabidopsis (Brophy et al., 2022) (**Figure 5A**). In this study, the authors developed a library of synthetic TFs capable of activating or repressing expression in a controlled, tissue-specific manner. By generating and leveraging synthetic promoters containing TFBSs recognized by synthetic regulators and placing them upstream of a *35S* minimal promoter, Brophy et al. constructed AND, OR, NOT, NAND, NOR, IMPLY, and NIMPLY logic gates to enable combinatorial control of gene expression. These gates were first validated in *N. benthamiana* leaves using GFP reporters before a subset of them were deployed in Arabidopsis stable transgenic lines. While showcasing the application of these gates, the authors were able to successfully modulate expression of the dominant *solitary root* (*slr*) mutant gene in Arabidopsis specifically within lateral root stem cells, resulting in precise alterations to lateral root density without affecting other root phenotypes, such as primary root growth or root hair density (Brophy et al., 2022). The authors highlighted that some of the logic gates required iterative optimization to achieve the desired spatial and temporal expression, underscoring both the challenges and the modularity inherent in synthetic promoter design. Two years later, another study by Khan et al. deployed a CRISPR interference (CRISPRi)-based gene circuit platform to construct reversible Boolean logic gates, particularly NOR, in *Arabidopsis* protoplasts and stably transformed plants (**Figure 5B**). These gates were then utilized to build complex logic circuits to confer NIMPLY and AND gate logic (**Figure 5B**). This work further demonstrated the utility of logic gates in programming desired, controlled plant responses by showing that combining gates, NOR gates in this case, to create circuits that impose logic functions that are generally more difficult to implement biologically (e.g., AND gates), can enable the rise of tighter, more specific, and non-leaky expression profiles (Khan et al., 2025). Both studies provide practical examples of genetic logic gates, the basic building blocks for the construction of complex synthetic circuits, functioning as intended *in vivo* to alter specific plant traits.

Building on this foundational work, Kong et al. established a predictive framework in plants to reliably generate and scale the development of promoter-driven genetic circuits in as little as 10 days (Kong et al., 2025) (**Figure 5C**). In this study, a synthetic cytokinin-inducible promoter *TCSn* and a native auxin-inducible *GH3.3* promoter were employed alongside orthogonal sensors and NOT logic gates to create a variety of genetic circuits capable of integrating two inputs (i.e., cytokinin trans-zeatin (tZ) and the synthetic auxin naphthaleneacetic acid (NAA)) to control the transcriptional output of a firefly luciferase (LUC) reporter. Synthetic promoters responsive to either auxin or cytokinin were utilized to drive expression of a transcriptional repressor designed to regulate *LUC*. The initial gate was designed to express the reporter gene only if neither NAA nor tZ were present by leveraging a transcriptional repressor (LmrA) to control *LUC* expression, creating a NOR gate (**Figure 5C**). This gate was validated in transient expression assays in Arabidopsis protoplasts. Simultaneously, a computational model was created to predict expression of this gate, and later other logic gates, under different input conditions (Kong et al., 2025). Various genetic circuits were tested transiently in Arabidopsis protoplasts in the presence and absence of repressor expression, where they behaved as predicted by the model, demonstrating the model’s high prediction accuracy. A subset of gates were adapted and implemented in stable Arabidopsis transgenic lines to test whether these gates could be utilized to reprogram Arabidopsis roots, with the NAND gate in particular showing strong suppression of reporter expression only when both inputs were present (**Figure 5C**). The authors then tested an OR gate *in vivo* to control cell death in *Nicotiana benthamiana*. They leveraged a *NUCLEOTIDE-BINDING LEUCINE-RICH REPEAT RECEPTOR* (*NLR*) gene driven by an inducible promoter to induce a plant hypersensitive response after exposure to either NAA or tZ (**Figure 5C**). Cell death was significantly higher when either or both inducers were present as compared to samples that had no added inducer, indicating that the OR gate was implemented successfully (Kong et al., 2025). This study serves as an indicator that promoter engineering is beginning to move beyond simple expression tuning and towards predictable phenotypic reprogramming.

A particularly exciting example of employing logic gates for controlled expression is a recent study that created a biosensor for phenylpropanoids, a class of plant metabolites (Ferreira and Antunes, 2024) (**Figure 5D**). Ferreira & Antunes created Boolean operators in plants that sense phenylpropanoid-related metabolite levels, implementing transcription-based AND, NAND, IMPLY and NIMPLY gates to control downstream expression in a biosensor system. This study used bacterial repressor allosteric TFs (aTFs) coupled with synthetic plant promoters that incorporate TFBS for aTFs and transcriptional activators to modulate gene activity in a ligand-specific manner, where levels of phenylpropanoid metabolites were leveraged as input to the genetic circuit (Ferreira and Antunes, 2024). In the default state of the biosensors created in this study, a constitutively expressed aTF binds to a response element (RE) in a promoter driving a firefly *LUC* gene, preventing reporter expression (**Figure 5D**). When phenylpropanoid metabolites are added, those metabolites bind to the aTF and prevent the TF from binding to its respective RE, therefore enabling *LUC* expression. Multiple aTF-metabolite pairs were integrated in various combinations and structures to create the Boolean operators, such as the NIMPLY circuit under which repression can be switched off with the addition of the phenylpropanoid metabolite naringenin, which were tested and validated in Arabidopsis protoplasts and *N. benthamiana* leaves (**Figure 5D**). By integrating logic gates with biosensors, researchers could program metabolic or signaling pathways to activate expression only under specific, defined conditions (Ferreira and Antunes, 2024).

### 7.4 Crop engineering

In plant biotechnology contexts, synthetic promoters have emerged as powerful tools for engineering crops and enhancing agronomic traits. Over the past five years, several notable examples have demonstrated how promoter design enables precise tuning of complex traits, from metabolic rewiring for yield improvement to inducible systems that provide targeted resistance against pests and pathogens. The following studies illustrate how synthetic promoters can be tailored to the unique challenges of crop engineering, offering fine-grained control over gene expression that traditional promoters cannot achieve.

One of the longest-standing applications of promoter engineering in crops is fruit-specific expression. Ripening is an ideal target for trait engineering in crops because it is highly consequential for crop quality. The tomato E8 and E4 promoters are classical examples and both are strongly induced during ripening in response to ethylene (Deikman et al., 1992; Xu et al., 1996). Researchers have leveraged these promoters to create a hybrid, synthetic promoter combination to drive ripening-stage expression of transgenes. According to a United States patent published in the year 2000, an E8–E4 hybrid promoter was designed and seemingly leveraged by commercial companies to reduce ethylene biosynthesis in specific fruit tissues (Bestwick and Kellogg, 2000). Though this patent has since expired, this hybrid promoter is a prime early example of commercial companies utilizing synthetic promoters for a direct application in crops.

The use of synthetic promoters also enables inducible metabolic production, in which plants can generate compounds of interest in a controlled manner. Forestier et al. demonstrated this in *Nicotiana benthamiana* using a heat-inducible promoter system to drive biosynthesis of casbene, a compound that is utilized in the pharmaceutical industry (Forestier et al., 2023). In plants containing a cassette of four casbene biosynthesis genes driven by a heat-inducible synthetic promoter, casbene accumulated substantially when the plant was exposed to elevated temperatures (40 °C for 2 hours) while expression was low in control conditions (22 °C) (Forestier et al., 2023). Although demonstrated in a model host, this system provides a proof-of-concept for the application of synthetic promoters to induce compound production or engineer metabolites in plants, showing how researchers could leverage this system to engineer crops to produce specialty metabolites on demand, thereby avoiding unintended consequences that may decrease crop viability.

Pathogen resistance traits offer another application for synthetic promoters in crop engineering. Sultana et al. identified CREs responsive to soybean cyst nematode (SCN) and constructed synthetic promoters, containing four copies of SCN-responsive CREs (i.e., *4×M1.1* and *4×M2.3* fused to a minimal *35S* core), that were active only during nematode infection (Sultana et al., 2022). In transgenic soybean assays, these promoters showed negligible background expression but strong induction at infection sites, particularly in root tissues, while showing no induction under abiotic stress (e.g., high salinity, drought, and cold) (Sultana et al., 2022). Similarly, another study engineered a synthetic pathogen-inducible promoter for potato, *2xS-4xD-NpCABE_core_*, incorporating tandem *cis*-elements fused to a core promoter from *Nicotiana plumbaginifolia* (Kauder et al., 2025). When used to drive expression of a modified version of the pathogen-inducible a virulence gene *Avr3a* in potato lines also containing the potato resistance gene *R3a* (as *R3a* is required for triggering cell death in cells that detect the *Avr3a* gene), the promoter conferred resistance to *Phytophthora infestans*, a causal agent of late blight, through programmed local cell death. The optimized version of the synthetic promoter provided robust inducibility while maintaining low basal activity under non-infected conditions, thereby avoiding mass cell death that may be associated with constitutive expression of *Avr3a* (Kauder et al., 2025). Aside from providing an application of synthetic promoters in the engineering of plant defense responses in crops, this study reinforces the design principles discussed earlier, that promoter performance depends on the choice and arrangement of CREs, as well as the minimal core promoter used.

In future endeavors, the scalability and effectiveness of crop engineering through the use of synthetic promoters will depend on the expansion of promoter libraries, systematically characterizing synthetic promoter performance in crops rather than model systems, and developing design rules for synthetic promoters in order to achieve predictable function across species. For a more extensive overview of synthetic promoter applications prior to 2023, see Yasmeen et al., which surveys promoter construction strategies and applications across various plant systems (Yasmeen et al., 2023).

## 8 Incorporation of computational approaches in promoter design

**Figure 6:**
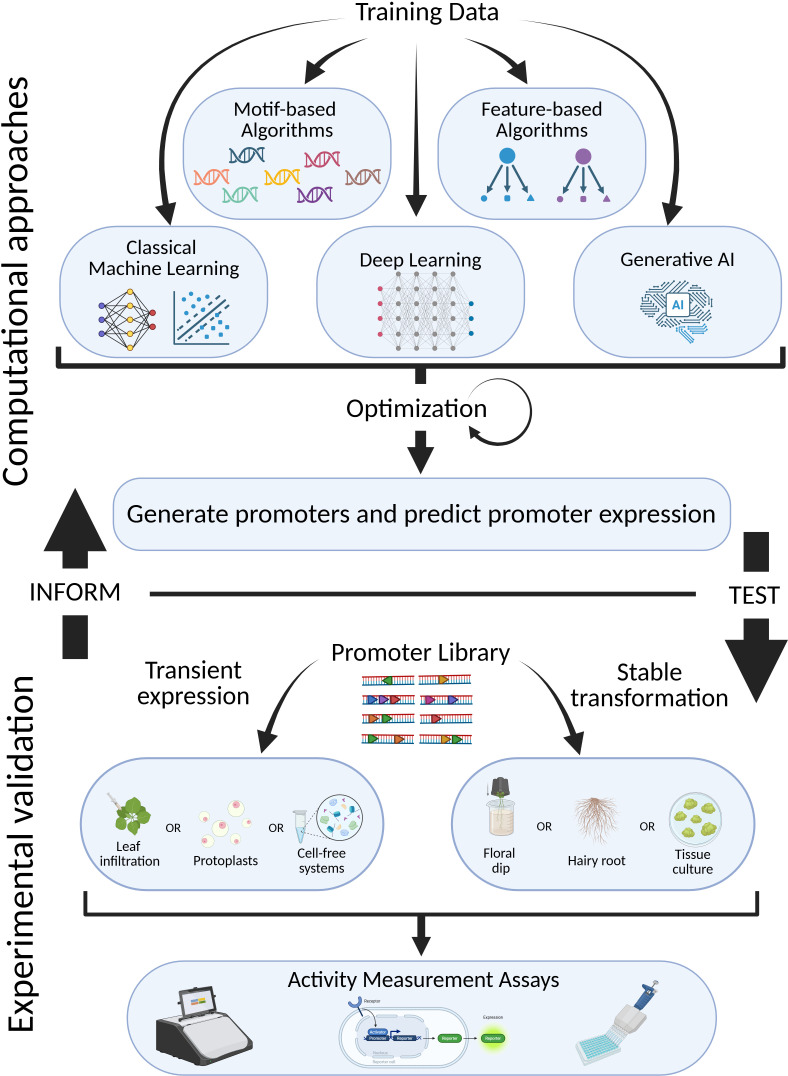
Illustration of the positive feedback loop between computational approaches and experimental validation. Computational approaches feed off of experimental data to train predictive or generative models. The promoters generated and their activity prediction levels are tested for accuracy via experimental validation, generally through transient expression or stable transformation approaches. The information gathered through activity measurement assays is then used to inform optimization and refinement of computational models, creating a positive feedback loop between computational and experimental approaches that leads to rational promoter design. Created in https://BioRender.com.

**Figure 7:**
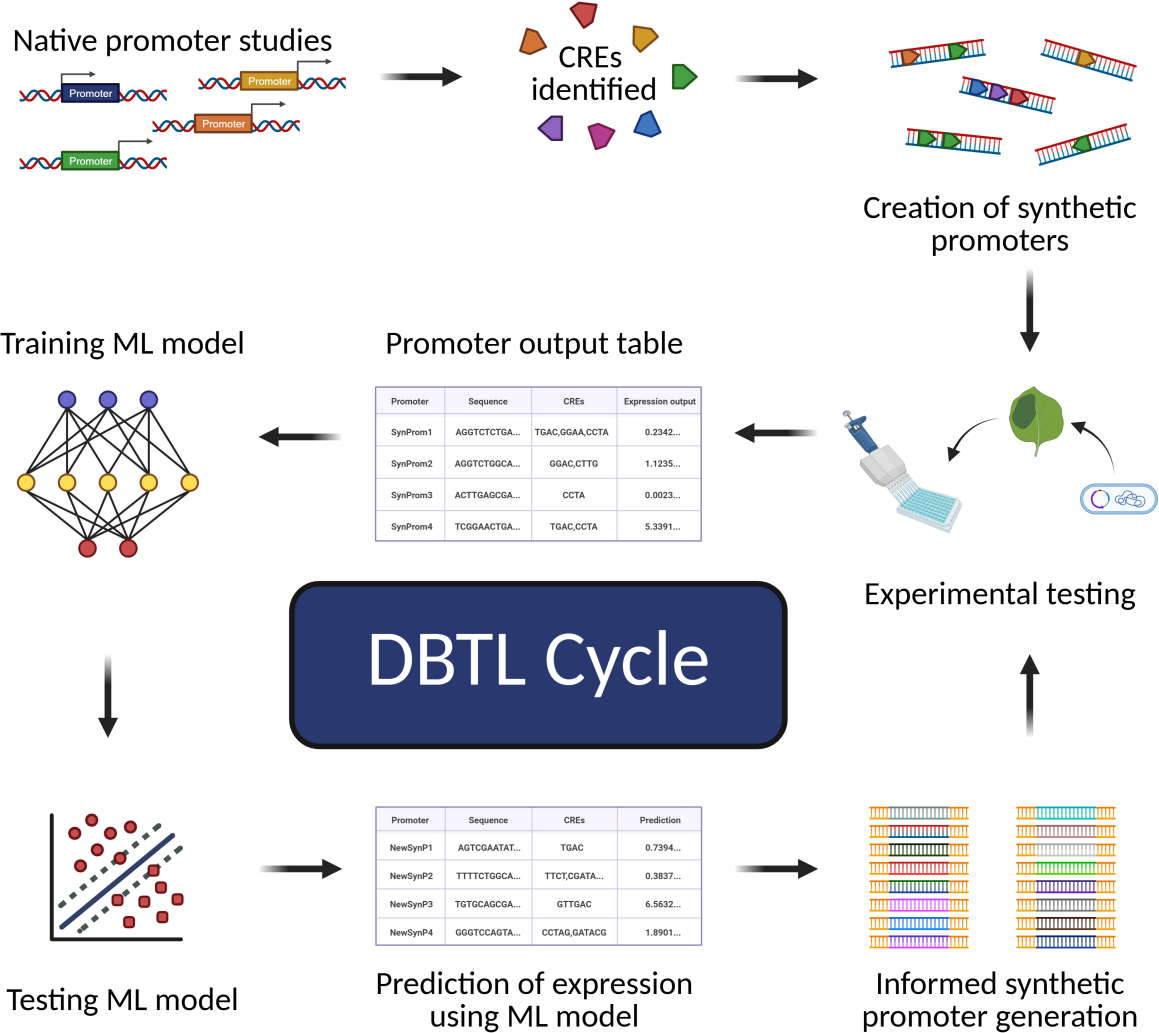
Schematic of the design-build-test-learn cycle. CREs used in the creation of synthetic promoters are typically identified and isolated from native promoter studies. Synthetic promoters are built and then tested experimentally using high-throughput quantitative assays, generating expression profiles or activity level values. Tables containing promoter information and reporter gene activation can be fed into ML models to train them on key features of synthetic promoter design. These ML models are then tested and utilized in the prediction of novel synthetic promoter designs. These informed designs are then tested experimentally, where the cycle begins again to continuously refine ML models. Created in https://BioRender.com.

As synthetic promoter design has progressed, computational methods have become increasingly central to furthering advancement, ranging from aiding in the discovery of CREs that underlie and define promoter function, to generating novel promoter sequences and predicting their behavior *in vivo.* Although a handful of plant-specific computational models exist (Deng et al., 2022; Peleke et al., 2024; Qiu et al., 2025), the most current models created for promoter engineering applications are still built and trained on data from prokaryotic, mammalian, or yeast species (Siwo et al., 2016; Fu et al., 2023; Seo et al., 2023; Gu et al., 2025). To provide a comprehensive view of how computational tools are being leveraged to advance synthetic promoter design, this section examines both plant-focused and non-plant computational approaches that could be adapted for plant-specific synthetic promoter design. We begin by reviewing computational strategies for CRE extraction and prediction, then assess sequence-based generation of synthetic promoters and predictive modeling of their expression levels, touching on the relationship between computational approaches and experimental validation (**Figure 6**). We then discuss efforts to incorporate chromatin and epigenetic features into CRE discovery and promoter activity prediction models, enabling a more realistic representation of the *in vivo* regulatory context. Finally, we wrap up this section with the importance of model optimization and iterative Design–Build–Test–Learn cycles that combine computational prediction and experimental data to identify high-performing promoters (Vollen et al., 2024) (**Figure 7**).

### 8.1 CRE extraction and prediction

As previously mentioned, the necessary first step in designing promoters is building a library of the short DNA motifs (TFBSs) that TFs recognize. Computational discovery of CREs has a long history and has benefited from both motif-discovery algorithms that utilize information gained from high-throughput plant TF-TFBS binding assays. Since their inception, motif discovery tools have established themselves as an integral part of CRE extraction. Early *in silico* prediction of CREs was limited due to a lack of known motifs across plant genomes (Rombauts et al., 2003). This limitation arose from the absence of sequencing data and centralized annotation databases of motifs rather than a lack of computational power and strategies, as algorithms developed in the early 2000s are still being leveraged decades later (Ho and Geisler, 2019). The classic Multiple Expectation maximizations for Motif Elicitation (MEME) Suite contains software tools like Find Individual Motif Occurrences (FIMO) that enable *de novo* motif discovery and motif scanning across promoters (Bailey et al., 2009; Grant et al., 2011). These tools are routinely used in plant research to identify which CREs are overrepresented in the promoters of co-regulated genes. For a more detailed review on using computational tools to identify CREs in plants, see the review by Zemlyanskaya et al., 2021 summarizing bioinformatics approaches that can be utilized to study transcriptional gene regulation (Zemlyanskaya et al., 2021).

Building on these foundational motif-discovery approaches, newer computational frameworks have begun to leverage deep learning (DL) to extract regulatory information directly from sequence data. iCREPCP is a DL-based platform that enables the identification of CREs within plant core promoters, trained on plant promoter activity datasets from Arabidopsis, maize, and tobacco (Deng et al., 2022) (**Table 2**). This program uses convolutional neural networks (CNNs) to identify individual bases or base pair windows that best inform promoter strength. CNNs can also aid in identifying proximal positional binding preferences of TFs, which can further inform where CREs should be placed within a promoter to promote gene activation (Rockenbach et al., 2025). By identifying the key sequence features that drive promoter activity, DL algorithms like iCREPCP can aid in guiding the design of synthetic promoters by streamlining CRE discovery in plants.

**Table 2:** Non-comprehensive compilation of machine learning methods developed for promoter design purposes.

Article	System/species	ML method(s)	Input	Output/task	Takeaway
iCREPCP (Deng et al., 2023)	*Arabidopsis thaliana, Sorghum bicolor*, and *Zea mays*	DenseNet (complex CNN)	Core promoter sequences	1. CRE identification 2. Promoter strength prediction	Web-based platform
Peleke et al., 2024	*Arabidopsis thaliana, Solanum lycopersicum, Sorghum bicolor*, and *Zea mays*	CNN	Upstream and downstream CDS- flanking regions (promoter + UTRs)	Gene expression prediction	Demonstrates the importance of UTRs in gene activity
Qiu et al., 2025	*Arabidopsis thaliana, Solanum lycopersicum, Oryza sativa*, and *Zea mays*	1. A plant-optimized Basenji2 model architecture (for CRE identification) 2. UMI-STARR-seq (for CRE strength prediction)	1. 3 kb (proximal) or 120 kb (distal) DNA sequence (in relation to a gene) 2. Commercially synthesized CREs	1. CRE identification 2. CRE strength prediction	Goal is to aid in promoter editing to alter gene expression predictably
Siwo et al., 2016	Yeast	SVM regression with SMO	1200 bp promoter sequences segmented into 100bp non-overlapping windows	Promoter strength prediction	Most predictive features were located 100bp upstream of the TSS
PromoDGDE (Gu et al., 2025)	*Escherichia coli* and *Saccharomyces cerevisiae*	Diffusion-GAN with reinforcement learning and evolutionary algorithm	Natural promoter sequences	Optimized synthetic promoters	Model that utilizes natural promoters to inform synthetic promoter generation
Seo et al., 2023	*Synechocystis* sp. PCC 6803 (cyanobacteria)	1. Generation: Variational Autoencoder 2. Prediction: CNN	1. Native promoter sequences 2. Synthetic promoter sequences	1. Synthetic promoter generation 2. Promoter strength prediction	Workflow could be adapted to plant species
DeePromoter (Oubounyt et al., 2019)	Human and mouse	CNN with long short-term memory	DNA sequence	Promoter vs non-promoter classification	Outperforms previous promoter classification methods
iPromoter-ET (Liang et al., 2021)	*Escherichia coli*	SVM after extremely randomized trees (ET) feature selection	DNA sequence	1. Promoter vs non-promoter classification 2. Promoter strength prediction	Outperforms existing related models for both classification and strength prediction
iProm-Zea (Kim et al., 2022)	*Zea mays*	Two-layer CNN	DNA sequence	1. TATA and TATA-less promoter prediction 2. Promoter strength prediction	Available as a free web tool
Fu et al., 2023	Human and mouse B-cells	CNN	B-cell-specific synthetic or natural promoters	Promoter strength prediction	Workflow could be adapted to plant species
GPro (Wang et al., 2024)	Any – demos available with yeast and *E.coli* data	Generative AI toolkit containing multiple models	Promoter sequence and sequence properties	Synthetic promoters	An open-source and user-friendly toolkit
Predmoter (Kindel et al., 2024)	Various plant species	Deep neural network (DNN)	Genomic DNA sequence	Prediction of ATAC- and ChIP-seq read coverage	Offers accurate cross-species base-wise prediction of genomic DNA accessibility
CharPlant (Shen et al., 2021)	*Arabidopsis thaliana, Solanum lycopersicum, Oryza sativa*, and *Medicago truncatula*	CNN	DNA sequence	Prediction of open chromatin regions	Capable of *de novo* prediction of open chromatin regions for a given DNA sequence

One recent study has extended the use of AI-based computational approaches beyond individual motif identification to the broader concept of creating compact enhancers for crop engineering (Yao et al., 2026). Yao et al. (2026) curated a set of almost 7000 natural short transcriptional enhancers (STEs) from maize, wheat, tomato, and soybean using a high-throughput screening platform, STEM-seq, which evaluated tens of thousands of candidate genomic elements and revealed thousands of functional enhancers with a wide range of activation levels. Building on this large data source, the authors developed BaseSearch, an AI-driven computational design framework that generated 5000 synthetic STE candidates, 455 of which were functional (1.0- to >2.0-fold activation), a success rate 11.4x higher than genome-wide screening alone could provide (Yao et al., 2026). Ten of those STEs exhibited very high activation, with the most active STE reaching 64.5-fold activation. These STEs were all, however, constitutive enhancers, with the authors noting that compact enhancers with spatiotemporal specificity may require more complex validation platforms, highlighting an existing limitation that can be explored further in future studies (Yao et al., 2026). By integrating high-throughput experimental data with AI-guided sequence design, this study exemplifies how computational modeling can accelerate the identification and creation of novel compact CREs for crop engineering.

### 8.2 Sequence-based promoter generation and predictive modeling

In contrast to CRE-focused methods, sequence-based approaches aim to establish predictive relationships between entire promoter sequences and total expression output. For example, Siwo and coauthors developed an algorithm to predict gene activity in yeast when given a particular DNA promoter sequence (Siwo et al., 2016) (**Table 2**). This algorithm was developed in response to an open community challenge by the Dialogue for Reverse Engineering Assessments and Methods (DREAM) consortium aiming to decipher the relationship between promoter sequence and gene expression. Particularly for this challenge, promoter sequences of yeast ribosomal protein (RP) genes were leveraged to understand the features in the promoter’s DNA sequence associated with expression patterns of a target gene. Since the promoter sequence was defined as the 1200bp upstream of a gene, the promoter region was segmented into 100bp non-overlapping windows to predict activity. The authors found success in utilizing support vector machine (SVM) regression to predict promoter activity along with sequential minimal optimization (SMO) to train the model, achieving a correlation coefficient of 0.65, higher than the other 20 teams that participated in the challenge. Though this model was originally developed and trained on yeast RP gene promoters, in the future, it could also be utilized to predict plant-specific promoter activity as long as there is a robust training set. Interestingly, Siwo et al.’s model focused solely on the 100bp region upstream of the gene rather than individual TFBSs in the promoter region. As such, the authors indicated that the incorporation of TF and nucleosome binding data into their model boosted the model’s predictive power, highlighting the importance of TFBS-independent feature identification in addition to traditional methods that focus on TFBS-based promoter activity prediction.

In the past few years, frameworks like Extended Vision Mutant Priority (EVMP) have been developed to strengthen machine learning (ML) models in prediction performance of promoter strength (Yang et al., 2023). These types of tools do not generally transfer well into plant applications, as the model requires high-throughput generation and characterization of synthetic promoter variants for its training dataset, a laborious process when conducted *in vivo* in plants. This bottleneck highlights the need for computational approaches that are plant-specific or more easily applicable to plant systems. Recent studies have begun addressing this gap by developing plant-specific DL tools. Peleke et al. developed a CNN to accurately predict gene expression profiles from gene flanking regions in Arabidopsis, tomato, sorghum, and maize (**Table 2**) (Peleke et al., 2024). The resulting DL model, which leveraged publicly available large-scale genomic data as its training dataset, achieved prediction accuracy exceeding 80%, relying only on upstream and downstream proximal *cis*-regulatory regions for prediction of gene expression profiles. Another study developed two DL models to predict expression of either long (120kb) or short (3kb) genomic sequences in maize, rice, tomato, or Arabidopsis in order to guide gene editing of promoters in plants (**Table 2**) (Qiu et al., 2025). These models allow for the identification of important CREs and prediction of their functional roles through *in silico* mutagenesis, which enables the prediction of expression changes associated with the point mutations made. Although these models are more geared towards regulating transcription through promoter editing in a genomic context, these algorithms can also possibly be utilized to gain more information on how to design a synthetic promoter to have desired patterns and levels of gene expression.

A model that is especially applicable to plants was developed by Washburn et al. in 2019 that sought to leverage DL to estimate relative gene transcript abundance by analyzing the associated DNA sequence (Washburn et al., 2019). Two models were created with both leveraging promoter and/or terminator sequences as input, one for single species applications to classify whether the gene expresses or not (called the pseudogene model) and the second to compare relative expression levels of two orthologous genes in multi-species applications (called the ortholog contrast model). These models were tested utilizing *Zea mays* and *Sorghum bicolor* data, with the pseudogene model reaching a total of 86.6% accuracy when both promoters and terminators from *Z. mays* were considered. The second model revealed various Pearson correlation coefficients depending on the source of ortholog pairs (i.e., comparing two *Z. mays* sub-genomes (0.59) or comparing *Z. mays* with *S. bicolor* (0.78)) (Washburn et al., 2019). These findings suggest that DL models leveraging promoter and/or terminator DNA sequences can capture informative features relevant to gene transcript abundance in plants, while also noting that species-specific factors and context may influence the model’s predictive performance. Applications of promoter strength prediction outside of plant research, particularly in mammalian studies, can provide some insight into the direction the plant researchers could take. Fu et al. developed a DL model to predict the transcriptional activity of B-cell specific promoters in human and mice (**Table 2**) (Fu et al., 2023). To train this CNN-based model, these authors computationally designed over 23,000 promoters by incorporating and shuffling around motifs from natural promoters driving Immunoglobulin genes into computationally generated background sequences (i.e., natural promoter sequences that were shuffled around). The promoters were designed by utilizing the MEME suite to identify motifs upstream of target genes and subsequently mapped to motif databases (e.g. JASPAR) (Bailey et al., 2009; Rauluseviciute et al., 2024). These synthetic promoters were then tested by massively parallel reporter assays to identify the features from promoter sequences that play a role in transcriptional regulation (Fu et al., 2023). The model was later used to predict the effect that polymorphisms in immunoglobulin V gene promoters would have on transcription of the gene across the global human population, leading to a better understanding of B-cell specific regulation mechanisms. The success of this pipeline highlights potential paths that researchers in the plant community can take to advance our understanding of plant-specific transcriptional mechanisms. By designing a targeted library of synthetic promoters, testing those promoters using massively parallel reporter assays, and feeding this large dataset into a DL model, we can accomplish multiple objectives at once: generate a diverse set of synthetic promoters with a targeted function for use in various applications; create a prediction model to accurately identify expression levels of a given promoter sequence; and deepen our understanding of how to engineer desired spatiotemporal patterns of expression accurately in plants.

Another study developed a generic framework based on DL methods that included a *de novo* synthetic promoter generation model alongside a transcriptional activity prediction model (**Table 2**) (Seo et al., 2023). A set of almost 7,000 cyanobacteria-derived natural promoters was utilized as a training set for the variational autoencoder (VAE)-based generative model to design 10,000 synthetic promoters for *Synechocystis* sp. PCC 6803, a model unicellular cyanobacterium (Seo et al., 2023). The promoters were then inputted into a CNN-based model to predict promoter strength and experimentally validated using a cell-free transcription assay. After refinement, the predictive model was able to reach a Pearson correlation coefficient of 0.57 in predicted versus tested transcriptional activity levels. Both the generative and predictive models were designed such that they could be implemented in other species, particularly in non-model organisms, by virtue of using a cell-free system to validate transcriptional activity (**Figure 6**). However, VAEs may be limited in their ability to generate long, diverse promoter sequences with accurately predicted levels of activity due to limitations in latent space, making VAEs more suitable for generating short promoter sequences (Wang et al., 2025). Similar workflows leveraging VAEs for promoter generation and CNNs for activity prediction could, in principle, be deployed directly for plant synthetic promoter engineering with minimal architectural changes. Thus, while not plant-trained, the study demonstrates an additional conceptual pathway for translating computational models developed in non-plant species into plant-specific synthetic promoter generation and validation.

To enhance generation of promoters with predictable expression and refine synthetic promoter engineering methods, we can turn our attention to computational approaches that focus on promoter classification and discrimination. Researchers have developed ML and DL models that classify given sequences into promoter or non-promoter categories, like DeePromoter and iProm-Zea (**Table 2**) (Kim et al., 2022; Oubounyt et al., 2019). One study trained computational models on either yeast, Arabidopsis, or human data, then evaluated them in a within-species and a cross-species manner to determine model performance (Bhandari et al., 2021). The observed decline in performance underscores the variation in promoter architectures across species, suggesting that these models should ideally be trained on data from the target species to ensure reliable predictions. Models like iPromoter-ET take this classification a step further, utilizing a two-layer predictor to first identify whether a sequence is a promoter or not, then determine the predicted strength of the promoter (**Table 2**) (Liang et al., 2021). Synthetic promoter design in plants can benefit from these types of models by feeding into them proposed synthetic promoter sequences and observing whether the model identifies the sequence as a promoter, as well as predicted promoter strength.

Combining both promoter generation with discrimination methods allows for the emergence of a framework that supports iterative model optimization. A recent study reported a novel promoter design method called PromoDGDE developed to address the need for intentional design of promoters with varying transcriptional strengths (Gu et al., 2025). This study combined an altered generative adversarial network (GAN) based method with an evolutionary algorithm and reinforcement learning to create optimized constitutively expressing synthetic promoters for *E. coli* and *Saccharomyces cerevisiae* (**Table 2**). Essentially, the GAN model is made up of two neural networks: a generator (G), that generates fake promoter sequences, and a discriminator (D), that discriminates between real promoters and the ones generated by the generator, in order to learn the true distribution of features in real promoter data. The synthetic promoters generated by this module are then fed into an optimization module leveraging evolutionary algorithm with reinforcement learning. Their transcriptional strength is predicted using a module that incorporates both a CNN and a long short-term memory (LSTM) network to help extract both local and long-range features that affect transcriptional activity, effectively increasing prediction accuracy. The prediction module allows for dynamic optimization of synthetic promoter design, as the optimization module can learn from the results of the prediction module through RL and, in turn, refine synthetic promoters to produce desired levels of expression. When tested *in vivo*, over 60% of the promoter sequences behaved as predicted, with a Pearson’s correlation coefficient of 0.79 and 0.9 for *E. coli* and *S. cerevisiae* respectively. However, this model does not take into consideration prior information and thus may fail to capture weak features during generation that may affect overall model performance. Despite this, the adaptive and optimization-oriented framework of PromoDGDE offers a compelling model for future efforts in plant synthetic promoter design. Theoretically, the plant research community could begin moving in this direction by tweaking optimization parameters and tailoring the training datasets to plant-specific constitutive promoters, then possibly narrow the training dataset to a particular promoter type for more specialized applications, as long as there is a large enough dataset to train the model without overfitting the data.

The integration of these diverse computational strategies with traditional experimental validation illustrates a shift in the advancement of promoter engineering (**Figure 6**). Studies in yeast have particularly highlighted the importance of this integration, with one DL model called CRMnet utilizing data from millions of experimentally validated promoters to pre-train the model before fine tuning (i.e. transfer learning) (Ding et al., 2023a; Dhaval Vaishnav et al., 2022; de Boer et al., 2020). Some recent studies in *E. coli* have even begun to move past activity prediction and towards generating target strength promoter sequences, showcasing the precision of DL models trained on large-scale data (Zhou et al., 2025). The successes achieved in non-plant systems can provide a blueprint for generative and predictive algorithms that accelerate the development of synthetic promoters with desired patterns and levels of expression. As plant-specific datasets grow, and model architectures become more transferable across species, these computational approaches are positioned to enable rational, guided, and efficient design of synthetic promoters in plants.

### 8.3 Models integrating chromatin and epigenetic information

In addition to promoter sequence-based activity prediction, considering the effects of epigenetic information can improve prediction accuracy of transcriptional output. In the modern day, chromatin accessibility assays such as ATAC-seq and DNase-seq are widely used in plants to annotate candidate regulatory regions and to differentiate motifs that fall within accessible versus not accessible sites in the tissue or conditions of interest. ATAC-seq has been utilized across various organs and cell types in Arabidopsis, maize, rice and other species to reveal both conserved (across species) and species-specific regulatory landscapes. Since promoter activity is correlated with local chromatin accessibility and histone modifications (e.g., H3K4me3), integrating ATAC-seq, ChIP-seq, and DAP-seq data into ML models that predict promoter design would greatly increase applicability of synthetic promoters that leverage native TFs. Models like CharPlant and Predmoter predict open chromatin or histone modifications directly from sequence, offering insights into where motifs are most likely to be functional (**Table 2**) (Kindel et al., 2024; Shen et al., 2021). These models are trained on DNase-seq, ChIP-seq, and/or ATAC-seq cross-species data and aim to narrow the search space of condition and tissue-relevant TF-TFBS pairs. Other types of models enable the integration of chromatin accessibility with CRE prediction by taking both a DNA sequence and a vector of epigenomic features as input and outputting expected promoter strength (Zhao et al., 2021). Motif enrichment in differential elements of accessibility (MEDEA) is a computational tool that evaluates high-throughput chromatin accessibility data to identify TFBS enrichment within accessible regions of the genome (Mariani et al., 2020). This helps identify biologically relevant TFBS that can be leveraged in synthetic promoters. Models that do not take into consideration epigenetic information will likely exhibit reduced predictive accuracy and produce promoters with limited generalizability across tissues, developmental stages, and environmental conditions. Tools incorporating chromatin accessibility are a useful resource for plant synthetic promoter design, as they can identify elements that look promising in sequence space but will be inaccessible due to blockage by heterochromatin in the tissue of a target species.

### 8.4 Optimization and iterative design-build-test-learn

In the context of promoter design, DBTL aims to develop potential promoters using computational modeling, build synthetic promoters or promoter libraries, test their activity experimentally, and learn from the data gathered by feeding it back to the model to improve predictions (**Figure 7**) (Vollen et al., 2024). This creates a recursive cycle of creating and testing synthetic promoters in an informed manner. To continue advancing the field, DBTL pipelines should be the next focus in plant promoter engineering (Wang and Demirer, 2023). Innovations such as high-throughput transient assays (e.g., protoplasts, leaf infiltration), massively parallel reporter assays, and improved DNA part assembly methods allow for rapid experimental testing of promoters, while computational approaches can be utilized to reduce the number of wet-lab experiments needed to identify synthetic promoters that function as expected. As previously mentioned, there has already been some work done to quantify the expression of synthetic part libraries using transient protoplast assays and dual-luciferase reporters, with transient characterization accurately predicting behavior in stable transformants (Schaumberg et al., 2016). These quantitative datasets make it possible to build trained models to inform promoter design based on predicted promoter activity, closing the DBTL loop by guiding experimental studies (i.e., representing the ‘Learn’ in DBTL). Over time, this iterative cycle continuously improves model performance, making it easier for researchers to construct synthetic promoters that show desired behavior in subsequent iterations.

One web-tool called PromoterCAD, created in 2013 but no longer available, utilized experimental data to inform data-mining tools that could identify CREs in Arabidopsis and provided a user interface (UI) for the design of synthetic promoters (Cox et al., 2013). PromoterCAD was a direct example of utilizing computational approaches to guide promoter design, representing the ‘design’ and ‘build’ parts of the DBTL cycle. In more recent years, GPro, a Generative Artificial Intelligence (AI) toolkit for promoter design, has been developed and made accessible to the public (**Table 2**) (Wang et al., 2024). This tool has been trained on both prokaryotic and eukaryotic sequences to generate synthetic promoter sequences and their predicted expression output. Additionally, the developers have documented an extensive wiki to enable researchers to customize and train the model for their specific needs, thus making it potentially useful in plant promoter design. This tool directly supports the DBTL framework, as the ability to customize the model allows researchers to revise the model based on experimental studies, representing the ‘learn’ part of the cycle.

Reddy et al. developed a workflow using conservative objective models (COMs) for model-based optimization (MBO) to discriminate between promoters specific to three different cell lines, highlighting a path to designing cell-type specific promoters (Reddy et al., 2024a). The models utilized were pretrained on large promoter-driven expression datasets from massively parallel reporter assays and then fine-tuned with a much smaller cell-specific promoter-driven expression dataset. The authors tested their model experimentally and found that their workflow was able to help redesign the promoters in the smaller promoter-driven expression dataset in a way that improved cell-specificity for more than 70% of sequences for two of the cell lines. The most important aspect of this workflow is that it can be implemented iteratively, following the DBTL cycle to improve cell-specificity of promoters with every iteration. As this workflow utilizes data from massively parallel reporter assays and was implemented using cell lines to represent different cell types, this may be difficult to translate into plants. There are no stable, fully differentiated plant cell lines that can be leveraged to refine cell-specificity of promoters to the degree that other fields may be able to given the availability of well-characterized animal cell lines. This is a challenge to keep in mind when translating this method to plant-specific applications, but it does not preclude the plant community from attempting to use the workflow to improve specificity of relevant promoters with currently existing plant cell-type specific data or utilizing fluorescence-sorted protoplasts from well-characterized cell-type-specific marker lines.

By utilizing iterative DBTL cycles for promoter engineering, researchers can progressively refine computational model performance and expand experimental libraries, as well as increase the accuracy of *in-silico* prediction with *in-planta* performance. Models like the one created by Seo and coauthors (Seo et al., 2023) showcase how computational methods can quickly generate a large library of novel synthetic promoters, but validation of activity predication is limited to current experimental approaches. On the other hand, the ability of computational approaches to accurately predict expression of a given promoter sequence is often limited by the availability of large training datasets. This is where large-scale experimental studies come into play, providing the bulk of data fed to computational models that enable the rational design of promoters (Qi et al., 2022; Jores et al., 2021; de Boer et al., 2020). These promoters, in turn, get built and tested experimentally (via transient or stable transformation) (**Figure 6**). The resulting validation data provide a feedback platform for computational models to refine predictions and improve future promoter designs, effectively closing the DBTL loop between computational and experimental approaches (**Figure 6**). Thus, DBTL approaches enable synthetic promoter design to go from a trial-and-error endeavor to a rational, predictive, and rapid workflow.

## 9 Challenges and future directions

Synthetic promoter design in plants has advanced rapidly over the past decade, thanks to improvements in high-throughput sequencing, computational modeling, and synthetic biology toolkits. However, significant challenges that limit the scalability, transferability, and predictability of promoter engineering remain. Despite the expansive repository of TF networks and known CREs, we are still lacking complete annotation of these relationships in most plant species. In most species, functional annotations of CREs are heavily biased toward orthologous genes from model organisms like Arabidopsis, maize, and rice. Current databases often rely on limited experimental datasets, requiring time-consuming manual curation and annotation. As noted, these databases generally lack information for non-model organisms, which stifles advancement of applied synthetic biology research. In polyploid crops that harbor extensive duplications of regulatory regions, designing synthetic promoters is especially challenging without detailed knowledge of TF–TFBS relationships. Emerging DL models are beginning to combat this by increasing robustness by training on data from polyploid species (Rockenbach et al., 2025). The integration of ChIP-seq, ATAC-seq, DAP-seq, and other previously mentioned sequencing techniques with ML and DL models is enabling high-throughput identification of CREs, greatly expanding our toolkit for synthetic promoter design.

However, current computational approaches still face limitations. Computational models rely on the identity of their training set which could lead to a couple of potential issues, including overfitting and non-transferability of the trained model. Additionally, models that are trained in one species often lose predictive power or fail when utilized in another species (Moore et al., 2020). This problem could theoretically be amplified when applying models trained on prokaryotic or mammalian data to generate plant-specific promoters, as there are differences in the contribution of DNA sequence features, like GC content or DNA structure, and epigenetic regulatory features, such as nucleosome placement or epigenetic marks (or lack thereof in the case of prokaryotes). Increasing generalizability of the model by increasing the range of species included in the training set may also decrease model performance if the dataset is not large enough, especially when using the model in a species-specific manner. Techniques like transfer learning, an ML technique where a trained model for a data-rich species is used as a starting point for a data-scarce species, could aid in increasing accuracy of promoter activity prediction models (Reddy et al., 2024b; Xia et al., 2024). Additionally, studies that generate synthetic promoters and predict their activity often only test a subset of those promoters due to limitations in high-throughput experimental validation methods (**Figure 6**) (Seo et al., 2023). These are all factors that must be taken into consideration when utilizing computational tools to inform promoter design.

Future progress in plant synthetic promoter design hinges on addressing the two challenges of data scarcity and biological complexity. To make further advancements, the field requires larger and more diverse training datasets (especially from crop species), standardization of synthetic promoter benchmarking, systematization of data collection from promoter characterization experiments in order to standardize input for computational models, and integration of computational frameworks that account for sequence structure, chromatin accessibility, and higher-order interactions. In addition to these measures, integrating high-throughput assays and the DBTL framework will advance the rational design of synthetic promoters and accelerate the creation of genetic logic gates that enable man-driven, tunable transcriptional regulation with exceptional precision in plants for both foundational and applied research. Moving forward, integrating systems biology, ML, and synthetic biology to advance plant promoter engineering will transform the field from a purely empirical discipline into an informed predictive science, enabling deeper insights into the fundamental logic of gene regulation in plants.
